# SARS-CoV-2 immune evasion by the B.1.427/B.1.429 variant of concern

**DOI:** 10.1126/science.abi7994

**Published:** 2021-08-06

**Authors:** Matthew McCallum, Jessica Bassi, Anna De Marco, Alex Chen, Alexandra C. Walls, Julia Di Iulio, M. Alejandra Tortorici, Mary-Jane Navarro, Chiara Silacci-Fregni, Christian Saliba, Kaitlin R. Sprouse, Maria Agostini, Dora Pinto, Katja Culap, Siro Bianchi, Stefano Jaconi, Elisabetta Cameroni, John E. Bowen, Sasha W. Tilles, Matteo Samuele Pizzuto, Sonja Bernasconi Guastalla, Giovanni Bona, Alessandra Franzetti Pellanda, Christian Garzoni, Wesley C. Van Voorhis, Laura E. Rosen, Gyorgy Snell, Amalio Telenti, Herbert W. Virgin, Luca Piccoli, Davide Corti, David Veesler

**Affiliations:** 1 Department of Biochemistry, University of Washington, Seattle, WA 98195, USA.; 2 Humabs Biomed SA, a subsidiary of Vir Biotechnology, 6500 Bellinzona, Switzerland.; 3 Vir Biotechnology, San Francisco, CA 94158, USA.; 4 Center for Emerging and Re-emerging Infectious Diseases, Division of Allergy and Infectious Diseases, Department of Medicine, University of Washington School of Medicine, Seattle, WA 98195, USA.; 5 Independent physician, 6828 Balerna, Switzerland.; 6 Clinical Research Unit, Clinica Luganese Moncucco, 6900 Lugano, Switzerland.; 7 Clinic of Internal Medicine and Infectious Diseases, Clinica Luganese Moncucco, 6900 Lugano, Switzerland.

## Abstract

A novel variant of concern (VOC) named CAL.20C (B.1.427/B.1.429), which was originally detected in California, carries spike glycoprotein mutations S13I in the signal peptide, W152C in the N-terminal domain (NTD), and L452R in the receptor-binding domain (RBD). Plasma from individuals vaccinated with a Wuhan-1 isolate–based messenger RNA vaccine or from convalescent individuals exhibited neutralizing titers that were reduced 2- to 3.5-fold against the B.1.427/B.1.429 variant relative to wild-type pseudoviruses. The L452R mutation reduced neutralizing activity in 14 of 34 RBD-specific monoclonal antibodies (mAbs). The S13I and W152C mutations resulted in total loss of neutralization for 10 of 10 NTD-specific mAbs because the NTD antigenic supersite was remodeled by a shift of the signal peptide cleavage site and the formation of a new disulfide bond, as revealed by mass spectrometry and structural studies.

## SARS-CoV-2 from alpha to epsilon

As battles to contain the COVID-19 pandemic continue, attention is focused on emerging variants of the severe acute respiratory syndrome coronavirus 2 (SARS-CoV-2) virus that have been deemed variants of concern because they are resistant to antibodies elicited by infection or vaccination or they increase transmissibility or disease severity. Three papers used functional and structural studies to explore how mutations in the viral spike protein affect its ability to infect host cells and to evade host immunity. Gobeil *et al*. looked at a variant spike protein involved in transmission between minks and humans, as well as the B1.1.7 (alpha), B.1.351 (beta), and P1 (gamma) spike variants; Cai *et al*. focused on the alpha and beta variants; and McCallum *et al*. discuss the properties of the spike protein from the B1.1.427/B.1.429 (epsilon) variant. Together, these papers show a balance among mutations that enhance stability, those that increase binding to the human receptor ACE2, and those that confer resistance to neutralizing antibodies. —VV

## Teaser

How SARS-CoV-2 variants gain enhanced infectivity and evade host immune responses.

COVID-19 is caused by severe acute respiratory syndrome coronavirus 2 (SARS-CoV-2) and is associated with acute respiratory distress syndrome, as well as extrapulmonary complications such as vascular thrombosis, coagulopathy, and a hyperinflammatory syndrome contributing to disease severity and mortality. SARS-CoV-2 infects target cells using the spike glycoprotein (S), which is organized as a homotrimer with each monomer comprising an S_1_ and an S_2_ subunit (*1*, *2*). The S_1_ subunit harbors the receptor-binding domain (RBD) and the N-terminal domain (NTD), as well as two other domains designated here as C and D (*3*, *4*). The RBD interacts with the angiotensin-converting enzyme 2 (ACE2) entry receptor on host cells through a subset of amino acids forming the receptor-binding motif (*1*, *2*, *5*–*7*). The NTD was suggested to bind DC-SIGN, L-SIGN, and AXL, which may act as attachment receptors (*8*, *9*). Both the RBD and the NTD are targeted by neutralizing antibodies (Abs) in infected or vaccinated individuals, and some RBD-specific monoclonal Abs (mAbs) are currently being evaluated in clinical trials or are authorized for use in COVID-19 patients (*10*–*24*). The S_2_ subunit is the fusion machinery that merges viral and host membranes to initiate infection and is the target of Abs cross-reacting with multiple coronavirus subgenera because of its higher sequence conservation compared with the S_1_ subunit (*25*–*28*).

The ongoing global spread of SARS-CoV-2 led to the fixation of the D614G substitution (*29*, *30*), as well as to the emergence of a large number of viral lineages worldwide, including several variants of concern (VOC). Specifically, the B.1.1.7, B.1.351, and P.1 lineages that originated in the United Kingdom, South Africa, and Brazil, respectively, are characterized by the accumulation of mutations in the S gene as well as in other genes (*31*–*33*). Some of these mutations lead to marked reductions in the neutralization potency of several mAbs, convalescent sera, and Pfizer/BioNTech BNT162b2– or Moderna mRNA-1273–elicited Abs (*19*, *34*–*40*). The B.1.1.7 variant has become dominant worldwide because of its higher transmissibility (*33*), underscoring the importance of studying and understanding the consequences of SARS-CoV-2 antigenic drift.

## Results

### The incidence of the B.1.427/B.1.429 lineages is increasing rapidly

The SARS-CoV-2 B.1.427/B.1.429 variant was reported for the first time at the beginning of 2021 in California, and as of May 2021 had been detected in 34 additional countries (*41*, *42*). The two lineages B.1.427 and B.1.429 (belonging to clade 20C according to Nextstrain designation) share the same S mutations (S13I in the signal peptide, W152C in the NTD, and L452R in the RBD) but harbor different mutations in other SARS-CoV-2 genes (*42*). Molecular clock analysis suggested that the progenitor of both lineages emerged in May 2020, diverging to give rise to the B.1.427 and B.1.429 lineages in June and July 2020 (*42*). The fast rise in the number of cases associated with the B.1.427/B.1.429 lineages led to their classification as a VOC by the US Centers for Disease Control and Prevention (https://www.cdc.gov/coronavirus/2019-ncov/cases-updates/variant-surveillance/variant-info.html).

**Fig. 1.**
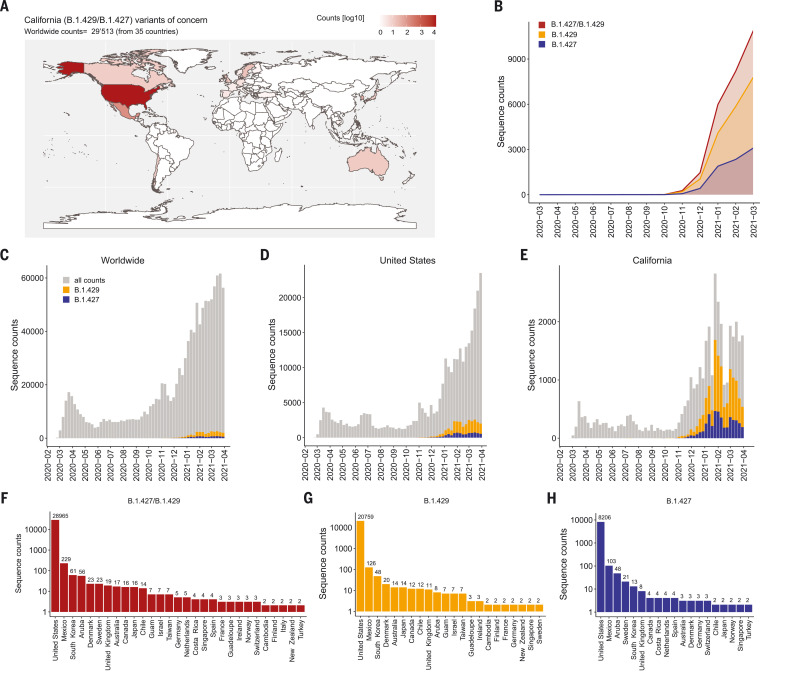
Geographic distribution and evolution of incidence over time of the SARS-CoV-2 B.1.427/B.1.429 VOC. (**A**) World map showing the geographic distribution and sequence counts of B.1.427/B.1.429 VOC as of 30 April 2021. (**B**) Cumulative and individual B.1.427/B.1.429 VOC sequence counts by month. (**C** to **E**) Total number of SARS-CoV-2 (gray) and B.1.427/B.1.429 VOC (blue/orange) sequences deposited on a monthly basis worldwide (C), in the United States (D), and in California (E). (**F** to **H**) Total number of B.1.427/B.1.429 (F), B.1.429 (G), and B.1.427 (H) sequences deposited by country as of 30 April 2021. Only countries with two or more deposited sequences are shown.

As of 30 April 2021, 8441 and 21,072 sequenced genomes had been reported in GISAID (Global Initiative for Sharing all Influenza Data) for the B.1.427 and B.1.429 lineages, respectively. This VOC was detected in California and in other US states, and more recently in 34 additional countries worldwide (Fig. 1, A to H, and table S1). The number of B.1.427/B.1.429 genome sequences deposited increased rapidly after December 2020, with an incidence exceeding 50% in California beginning in February 2021 (Fig. 1, B to E). Collectively, this analysis illustrates the increased incidence of the B.1.427/B.1.429 VOC and its progressive geographical spread from California to other US states and other countries, which is consistent with a recent study suggesting enhanced transmissibility relative to the ancestral isolate (*42*).

### B.1.427/ B.1.429 S reduces sensitivity to vaccine-elicited Abs

**Fig. 2.**
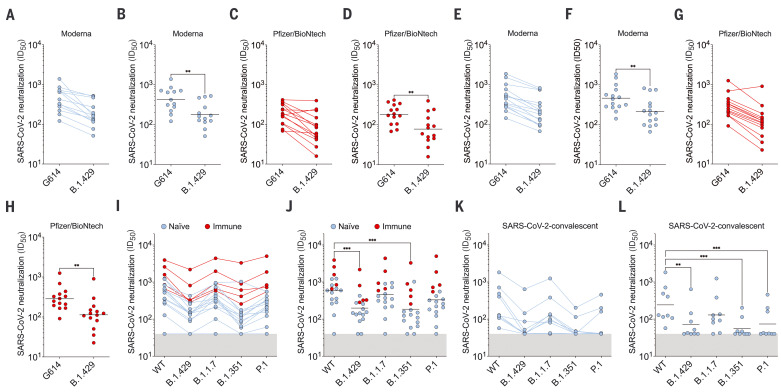
B.1.427/B.1.429 S pseudotyped virus neutralization by vaccine-elicited and COVID-19 convalescent plasma. (**A**, **B**, **E**, and **F**) Neutralizing Ab titers [mean inhibition dilution (ID_50_)] shown as pairwise connected [(A) and (E)] or GMT [(B) and (F)] against MLV [(A) and (B)] or VSV [(E) and (F)] pseudotyped viruses harboring G614 SARS-CoV-2 S or B.1.427/B.1.429 (B.1.429) S determined using plasma from individuals who received two doses of the Moderna mRNA-1273 vaccine (blue). (**C**, **D**, **G**, and **H**) Neutralizing Ab titers (ID_50_) shown as pairwise connected [(C) and (G)] or GMT [(D) and (H)] against MLV [(C) and (D)] or VSV [(G) and (H)] pseudotyped viruses harboring G614 SARS-CoV-2 S or B.1.427/B.1.429 (B.1.429) S determined using plasma from individuals who received two doses of the Pfizer/BioNtech BNT162b2 mRNA vaccine (red). (**I** and **J**) Neutralizing Ab ID_50_ (I) and GMT (J) titers against VSV pseudotyped viruses harboring D614 SARS-CoV-2 S, B.1.427/B.1.429 S, B.1.1.7 S, B.1.351 S, or P.1 S determined using plasma from naïve (blue) and previously infected (red) individuals who received two doses of the Pfizer/BioNtech BNT162b2 mRNA vaccine. “Naïve” indicates vaccinated individuals who had not been previously infected with SARS-CoV-2; “immune” refers to vaccinated individuals who had been previously infected with SARS-CoV-2. (**K** and **L**) Neutralizing Ab ID_50_ (K) and GMT (L) titers against VSV pseudotyped viruses harboring D614 SARS-CoV-2 S, B.1.427/B.1.429 S, B.1.1.7 S, B.1.351 S, or P.1 S determined using plasma from convalescent individuals who were infected with WT SARS-CoV-2. Neutralization data shown in (A) to (H) and (I) to (L) were performed using 293T-ACE2 and VeroE6-TMPRSS2, respectively. Data are average of *n = 2* replicates.

To assess the impact of the three mutations present in the B.1.427/B.1.429 S glycoprotein on neutralization, we first compared side by side the neutralization potency of mRNA vaccine–elicited Abs against the G614 S and B.1.427/B.1.429 S pseudoviruses. We used plasma from 15 individuals who received two doses of the Moderna mRNA-1273 vaccine and from 15 individuals who received two doses of Pfizer/BioNtech BNT162b2 vaccine collected between 7 and 27 days after booster immunization (table S2). All vaccinees had substantial plasma-neutralizing activity against G614 SARS-CoV-2 S pseudotyped viruses. Using a murine leukemia virus (MLV) pseudotyping system, geometric mean titers (GMTs) showed that the average neutralization potency of the Moderna mRNA1273–elicited plasma was reduced 2.4-fold for B.1.427/B.1.429 S (GMT: 178) compared with G614 S (GMT: 424) (Fig. 2, A and B; figs. S1 and S2; and table S3), whereas it was reduced 2.3-fold with Pfizer/BioNtech BNT162b2–elicited plasma (B.1.427/B.1.429 GMT: 78 versus G614 GMT: 182) (Fig. 2, C and D; figs. S1 and S2; and table S3). Using a vesicular stomatitis virus (VSV) pseudotyping system, we observed a 2.2-fold average reduction of Moderna mRNA1273–elicited plasma-neutralizing activity against B.1.427/B.1.429 S (GMT: 213) compared with G614 S (GMT: 464) pseudoviruses (Fig. 2, E and F; figs. S1 and S2; and table S3) and a 2.5-fold average reduction of Pfizer/BioNtech BNT162b2–elicited plasma-neutralizing activity against B.1.427/B.1.429 S (GMT: 113) compared with G614 S (GMT: 285) pseudoviruses (Fig. 2, G and H; figs. S1 and S2; and table S3). We also analyzed plasma from 18 individuals, five of whom were previously infected with wild-type (WT) SARS-CoV-2, who received two doses of the Pfizer/BioNtech BNT162b2 vaccine and whose samples were collected between 14 and 28 days after booster immunization. We compared the neutralization potency of Pfizer/BioNtech BNT162b2 vaccine–elicited Abs against D614 S, B.1.427/B.1.429 S, B.1.1.7 S, B.1.351 S, and P.1 S VSV pseudotyped viruses using Vero E6 expressing TMPRSS2 as target cells. GMT plasma neutralization potency was reduced 2.9-fold for B.1.427/B.1.429 S (GMT: 197) compared with D614 S (GMT: 570), which is a decrease comparable to that observed with B.1.351 (GMT: 180, 3.2-fold reduction) and greater than that observed with B.1.1.7 and P.1 (GMT: 450 and 330, 1.3-fold and 1.7-fold reduction, respectively) pseudotyped viruses (Fig. 2, I and J; figs. S1 and S2; and table S3). These data indicate that the three B.1.427/B.1.429 S residue substitutions led to a modest but significant reduction of neutralization potency from vaccine-elicited Abs.

We also analyzed plasma from nine convalescent donors who experienced symptomatic COVID-19 in early 2020 (and consequently were likely exposed to the Wuhan-1 or a closely related SARS-CoV-2 isolate) collected 15 to 28 days after symptom onset (table S2). The neutralization potency of plasma from the nine convalescent donors was reduced 3.4-fold for B.1.427/B.1.429 S (GMT: 70) compared with G614 S (GMT: 240), similar to what we observed with B.1.351 (4.4-fold, GMT: 55) and P.1 (3.3-fold, GMT: 72) pseudotyped viruses, whereas neutralization of B.1.1.7 was less affected (1.9-fold, GMT: 127) (Fig. 2, K and L; figs. S1 and S2; and table S3). In several cases, the level of neutralizing activity against the VOC was found to be below the limit of detection.

These findings show that the three mutations present in the B1.427/B.1.429 S glycoprotein decrease the neutralizing activity of vaccine-elicited and infection-elicited Abs, suggesting that these lineage-defining residue substitutions are associated with immune evasion. However, these data also underscore the higher quality of Ab responses induced by vaccination compared with infection and their enhanced resilience to mutations found in VOC.

### B.1.427/B.1.429 S mutations reduce sensitivity to RBD- and NTD-specific Abs

To evaluate the contribution of RBD and NTD substitutions to the reduced neutralization potency of sera from vaccinees and convalescent plasma, we compared the neutralizing activity of 34 RBD and 10 NTD mAbs against the D614 S or B.1.427/B.1.429 S variant using a VSV pseudotyping system (*1*, *43*).

**Fig. 3.**
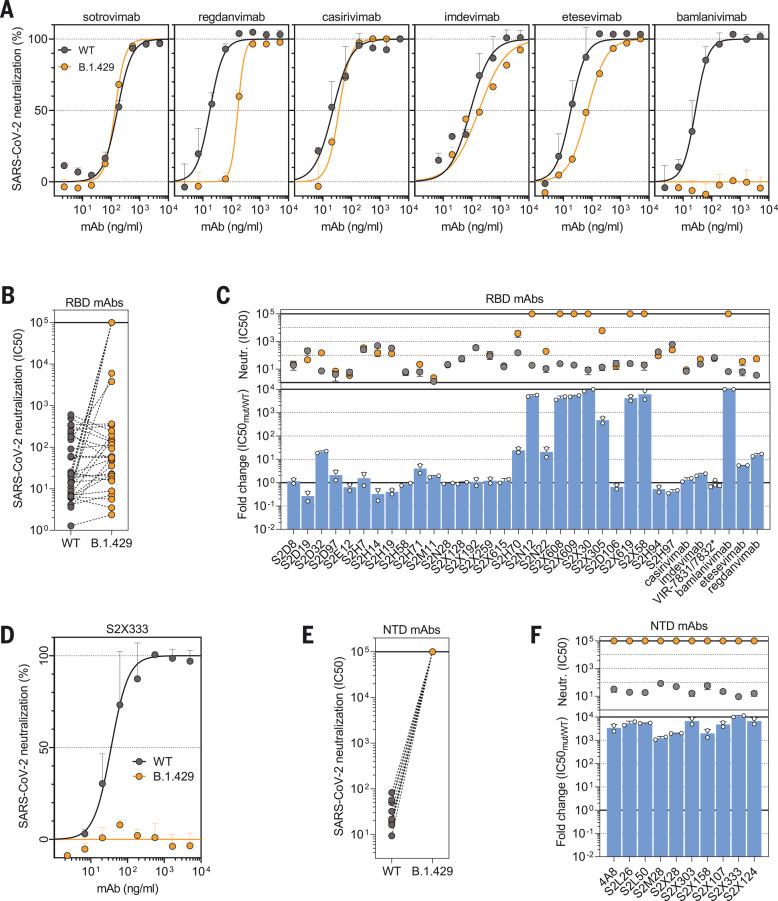
Neutralization by a panel of RBD- and NTD-specific mAbs against SARS-CoV-2 D614 S and B.1.427/B.1.429 S pseudoviruses. (**A** and **D**) Neutralization of SARS-CoV-2 pseudotyped VSV carrying D614 (gray) or B.1.427/B.1.429 (orange) S protein by clinical stage RBD mAbs (A) and an NTD-targeting mAb (S2X333) (D). Data are representative of *n* = 2 replicates. (**B** and **E**) Neutralization of SARS-CoV-2 S VSV pseudotypes carrying D614 or B.1.427/B.1.429 S by 34 mAbs targeting the RBD (B) and 10 mAbs targeting the NTD (E). Data are the mean of 50% inhibitory concentration (IC_50_) values (in nanograms per milliliter) of *n* = 2 independent experiments. Non-neutralizing IC_50_ titers were set at 10^5^ ng/ml. (**C** and **F**) Neutralization by RBD-specific (C) and NTD-specific (F) mAbs shown as mean IC_50_ values (top) and mean fold change (bottom) for B.1.427/B.1.429 S (orange) relative to D614 S (gray) VSV pseudoviruses. VIR-7831 is a derivative of S309 mAb (sotrovimab). *VIR-7832 (variant of VIR-7831 carrying the LS-GAALIE Fc mutations) shown as squares. Non-neutralizing IC_50_ titers and fold change were set to 10^5^ ng/ml and 10^4^, respectively.

The panel of RBD-specific mAbs (including six clinical mAbs) recognizes distinct antigenic sites, as previously characterized (*10*, *11*, *13*, *20*, *44*, *45*). Briefly, epitopes span the receptor-binding motif (antigenic sites Ia and Ib), a cryptic antigenic site II, the exposed N343 glycan–containing antigenic site IV, and a second cryptic antigenic site V (*10*, *11*). A total of 14 out of 34 mAbs showed a reduced neutralization potency when comparing B.1.427/B.1.429 S and D614 S pseudoviruses (Fig. 3, A to C, and fig. S3). Of the six mAbs in clinical use, regdanvimab (CT-P59), and to a smaller extent etesevimab (LY-CoV016), showed a reduction in neutralization potency, whereas bamlanivimab (LY-CoV555) entirely lost its neutralizing activity. Neutralization mediated by the casirivimab/imdevimab mAb cocktail (REGN10933 and REGN10987) (*14*, *15*) and by VIR-7831 (a derivative of S309, recently renamed sotrovimab) (*10*, *23*, *24*) was unaffected by the B.1.427/B.1.429 S variant. To address the role of the L452R mutation in the neutralization escape from RBD-specific Abs, we tested the binding of the 34 RBD-specific mAbs to WT and L452R mutant RBD using biolayer interferometry (fig. S4). The 10 RBD-specific mAbs experiencing a 10-fold or greater reduction in neutralization potency of the B.1.427/B.1.429 variant relative to D614 S bound poorly to the L452R RBD mutant, demonstrating a direct role of this mutation in immune evasion.

The neutralizing activity of all 10 NTD-specific mAbs tested was abolished as a result of the presence of the S13I and W152C mutations (Fig. 3, D to F). These data indicate that the decreased potency of neutralization of the B.1.427/B.1.429 variant results from evasion of both RBD- and NTD-specific mAb-mediated neutralization.

### Structural characterization of the SARS-CoV-2 B.1.427/B.1.429 S trimer

**Fig. 4.**
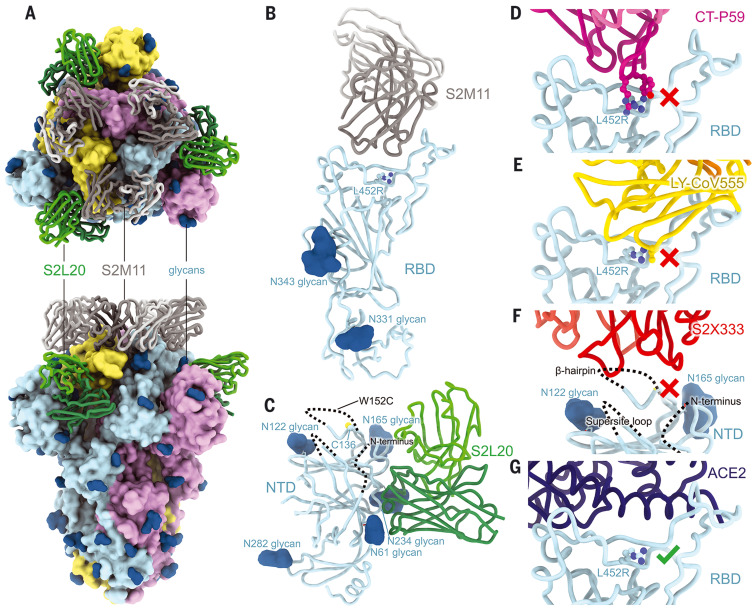
CryoEM structure of the SARS-CoV-2 B.1.427/B.1.429 S ectodomain trimer. (**A**) Structure of the S trimer (surface rendering) bound to the S2M11 and S2L20 Fabs (ribbons) in two orthogonal orientations. SARS-CoV-2 S protomers are colored pink, cyan, and gold, and the S2L20 Fab heavy and light chains are colored dark and light green, respectively, and the S2M11 Fab heavy and light chains are colored dark and light gray, respectively. Only the Fab-variable domains are resolved in the map. N-linked glycans are rendered as dark blue spheres. (**B**) Magnified view of the S2M11-bound RBD with R452 shown in ball and stick representation. (**C**) Magnified view of the S2L20-bound NTD with disordered N terminus, supersite β-hairpin, and loop regions shown as dashed lines. (**D**) Superimposition of the CT-P59–bound SARS-CoV-2 RBD structure (PDB 7CM4) on the SARS-CoV-2 B.1.427/B.1.429 S cryoEM structure showing that R452 would sterically clash with the mAb. (**E**) Superimposition of the LY-CoV555–bound SARS-CoV-2 RBD structure (PDB 7KMG) on the SARS-CoV-2 B.1.427/B.1.429 S cryoEM structure showing that L452R would sterically clash with the mAb. (**F**) Superimposition of the S2X333-bound SARS-CoV-2 S structure (PDB 7LXW) on the SARS-CoV-2 B.1.427/B.1.429 S cryoEM structure showing that most of the NTD antigenic supersite epitope residues are disordered. (**G**) Superimposition of the ACE2-bound SARS-CoV-2 RBD structure (PDB 7DMU) on the SARS-CoV-2 B.1.427/B.1.429 S cryoEM structure showing that L452R points away from the interface with ACE2.

To visualize the changes in SARS-CoV-2 B.1.427/B.1.429 S that contribute to immune evasion, we determined the cryo–electron microscopy (cryoEM) structure of the variant S ectodomain trimer [carrying the HexaPro mutations (*46*)] bound to the RBD-specific mAb S2M11 and the NTD-specific mAb S2L20 at 2.3-Å resolution (Fig. 4A, fig. S5, and table S4). S2M11 was used to lock the RBDs in the closed state (Fig. 4B), and S2L20 was used to stabilize the NTDs (Fig. 4C) (*12*, *13*). Superimposing the regdanvimab (CT-P59)–bound and bamlanivimab (LY-CoV555)–bound SARS-CoV-2 RBD structures to B.1.427/B.1.429 S revealed that the introduced L452R is sterically incompatible with binding of these mAbs (Fig. 4, D and E), rationalizing the dampening or loss of neutralizing activity.

We subsequently used local refinement to account for the conformational dynamics of the NTD and S2L20 relative to the rest of S and obtained a cryoEM reconstruction of the NTD bound to S2L20 at 3.0-Å resolution (Fig. 4C, fig. S5, and table S4). The structure revealed that the B.1.427/B.1.429 NTD antigenic supersite is severely altered. The N terminus is disordered up to residue 27, as is the supersite β-hairpin (disordered between residues 137 and 158) and the supersite loop (disordered between residues 243 and 264) (Fig. 4F). These structural changes explain the abrogation of binding and neutralization of the panel of NTD-specific mAbs evaluated.

Overlaying an ACE2-bound SARS-CoV-2 RBD structure with the B.1.427/B.1.429 variant S structure showed that the R452 residue points away from and does not contact ACE2, suggesting that this substitution would not affect receptor engagement (Fig. 4G). We next evaluated binding of the monomeric human ACE2 ectodomain to immobilized B.1.427/B.1.429 and WT RBDs using surface plasmon resonance (fig. S6, A and B, and table S5); biolayer interferometry (fig. S6, C to E, and table S5); and binding of B.1.427/B.1.429, B.1.1.7, and WT RBDs to immobilized human ACE2 by enzyme-linked immunosorbent assay (ELISA) (fig. S6F and table SS5). Our results indicate that the B.1.427/B.1.429 and WT RBDs bound to ACE2 with comparable affinities, whereas the B.1.1.7 RBD had a markedly increased affinity for ACE2 (*34*), validating the structural observations.

### Disulfide bond rearrangement in the B.1.427/B.1.429 variant NTD antigenic supersite

**Fig. 5.**
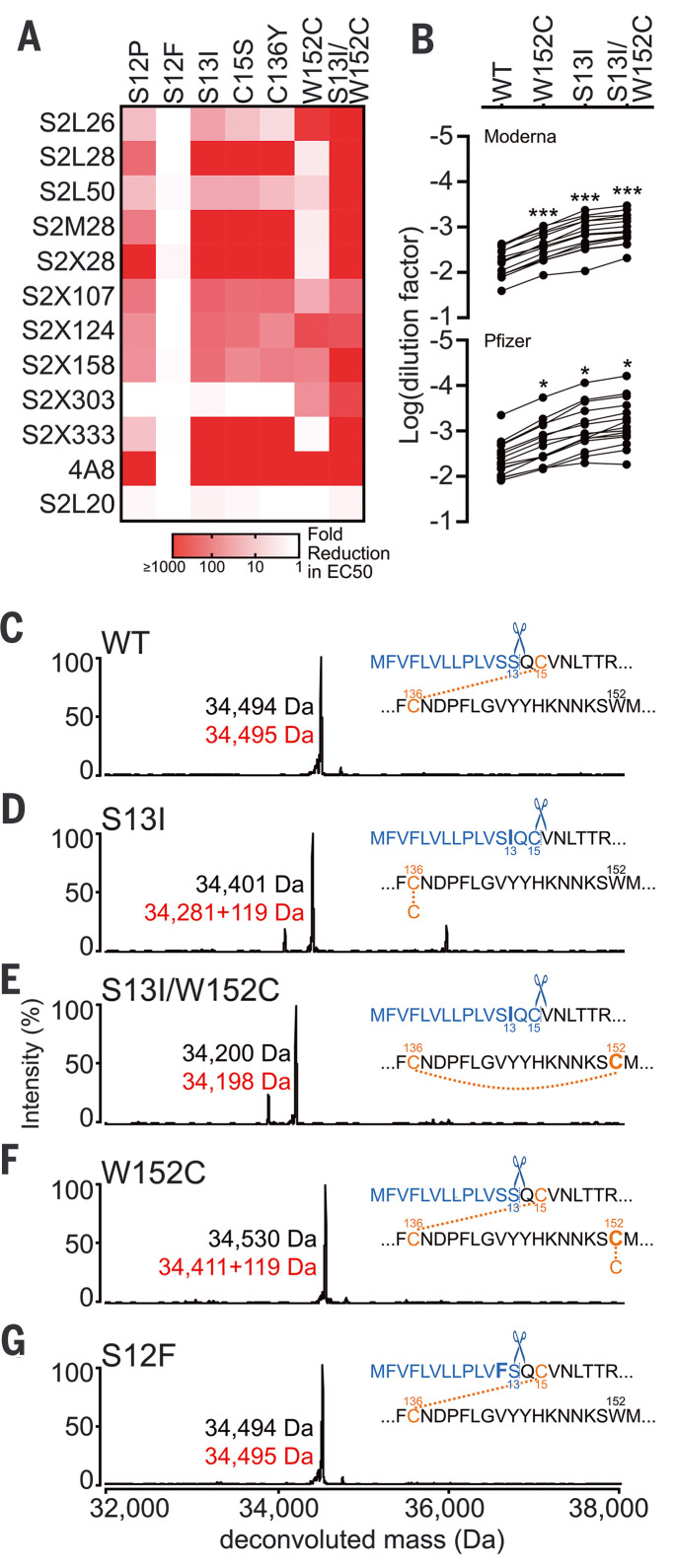
The B.1.427/B.1.429 S S13I and W152C mutations lead to immune evasion. (**A**) Binding of a panel of 11 neutralizing (antigenic site i) and one non-neutralizing (antigenic site iv) NTD-specific mAbs to recombinant SARS-CoV-2 NTD variants analyzed by ELISA displayed as a heatmap. (**B**) Binding of plasma Abs from vaccinated individuals to recombinant SARS-CoV-2 NTD variants analyzed by ELISA. The mean dilution factor for each mutant was compared by the one-way ANOVA test against WT (*P < 0.05, **P < 0.001). (**C** to **G**) Deconvoluted mass spectra of purified NTD constructs, including the WT NTD with the native signal peptide (C), the S13I NTD (D), the S13I and W152C NTD (E), the W152C NTD (F), and the S12F NTD (G). The empirical mass (black) and theoretical mass (red) are shown beside the corresponding peak. An additional 119 Da were observed for the S13I and W152C NTDs, corresponding to cysteinylation of the free cysteine residue in these constructs (as L-cysteine was present in the expression media). The cleaved signal peptide (blue text) and subsequent residue sequence (black text) are also shown based on the MS results. Mutated residues are shown in bold. Cysteines are highlighted in light orange (unless in the cleaved signal peptide), and disulfide bonds are shown as dotted light orange lines between cysteines. Residues are numbered for reference.

To investigate further the molecular basis for the loss of NTD-directed mAb-neutralizing activity and structural changes in the NTD, we analyzed the binding of a panel of NTD-specific mAbs to recombinant SARS-CoV-2 NTD variants using ELISA. The S13I signal peptide mutation dampened binding of five mAbs and abrogated binding of five additional mAbs of the 11 neutralizing mAbs evaluated (Fig. 5A and fig. S7). Furthermore, the W152C mutation reduced recognition of six NTD-neutralizing mAbs, including a complete loss of binding for two of them, with a pattern complementary to that observed for S13I (Fig. 5A and fig. S7). The B.1.427/B.1.429 S13I/W152C NTD did not bind to any NTD-directed neutralizing mAbs, which are known to target a single antigenic site (antigenic site I) (*12*), whereas binding of the non-neutralizing S2L20 mAb to the NTD antigenic site IV was not affected by any mutants, confirming proper retention of folding, as supported by the structural data (Fig. 5A and fig. S7). Binding of vaccine-elicited plasma to NTD mutants confirmed and extended these observations with polyclonal Abs by showing an increasingly marked reduction in binding titers caused by the W152C, S13I, and S13I/W152C residue substitutions (Fig. 5B and fig. S8).

We previously showed that disruption of the C15/C136 disulfide bond that connects the N terminus to the rest of the NTD by mutation of either residue or alteration of the signal peptide cleavage site abrogates the neutralizing activity of mAbs targeting the NTD antigenic supersite (site I) (*12*). Because the S13I substitution resides in the signal peptide and is predicted to shift the signal peptide cleavage site from S13-Q14 to C15-V16, we hypothesized that this substitution indirectly affects the integrity of NTD antigenic site I, which comprises the N terminus. Mass spectrometry analysis of the S13I and S13I/W152C NTD variants confirmed that signal peptide cleavage occurs immediately after residue C15 (Fig. 5, C to E). As a result, C136, which would otherwise be disulfide linked to C15, is cysteinylated in the S13I NTD because of the presence of free cysteine in the expression medium (Fig. 5D and fig. S9). Likewise, the W152C mutation, which introduces a free cysteine, was also found to be cysteinylated in the W152C NTD (Fig. 5F). It is not clear whether cysteinylation would occur during natural infection with S13I or W152C mutants alone or what contribution cysteinylation plays in immune evasion of S13I or W152C mutants alone. Dampening of NTD-specific neutralizing mAb binding is stronger for the S13I mutant than for the S12P mutant, which we previously showed also shifts the signal peptide cleavage site to C15-V16 (Fig. 5A). Conversely, we did not observe any effect on mAb binding of the S12F substitution, which has also been detected in clinical isolates, in agreement with the fact that this mutation did not affect the native signal peptide cleavage site (i.e., it occurs at the S13-Q14 position), as observed by mass spectrometry (Fig. 5G). In the absence of the C15-C136 disulfide bond, the N terminus is no longer stapled to the NTD, consistent with the structural data showing that the N terminus of the B.1.427/B.1.429 variant becomes disordered relative to the rest of the NTD (Fig. 4C).

Although the S13I and W152C NTD variants were respectively cysteinylated at positions C136 and W152C, the double mutant S13I/W152C was not cysteinylated, suggesting that C136 and W152C had formed a new disulfide bond (Fig. 5, D to F). Tandem mass spectrometry analysis of nonreduced, digested peptides identified linked discontinuous peptides containing C136 and W152C (fig. S9), confirming that a disulfide bond forms between C136 and W152C in the S13I/W152C NTD of the B.1.427/B.1.429 variant. W152C is in the β-hairpin of the antigenic supersite, and the formation of a new disulfide bond with C136 would move residues in the β-hairpin >20 Å. The local structure of the β-hairpin was disordered in the B.1.427/B.1.429 variant (Fig. 4C).

Collectively, these findings demonstrate that the S13I and W152C mutations found in the B.1.427/B.1.429 S variant are jointly responsible for escape from NTD-specific mAbs because of deletion of the two SARS-CoV-2 S N-terminal residues and overall rearrangement of the NTD antigenic supersite. Our data support that the SARS-CoV-2 NTD evolved a compensatory mechanism to form an alternative disulfide bond and that mutations of the S signal peptide occur in vivo in a clinical setting to promote immune evasion. The SARS-CoV-2 B.1.427/B.1.429 S variant therefore relies on an indirect and unusual neutralization escape strategy.

## Discussion

Serum- or plasma-neutralizing activity is a correlate of protection against SARS-CoV-2 challenge in nonhuman primates (*47*, *48*), and treatment with several neutralizing mAbs has reduced viral burden and decreased hospitalization and mortality in clinical trials (*10*, *14*, *15*, *22*, *23*, *49*). The observed L452R-mediated immune evasion of B.1.427/B.1.429 S is consistent with previous findings showing that this substitution reduced the binding or neutralizing activity of some mAbs before the discovery of the B.1.427/B.1.429 variant (*50*–*53*). The acquisition of the L452R substitution by multiple lineages across multiple continents, including the B.1.617.1 and B.1.617.2 lineages emerging in India (*54*), is suggestive of positive selection, which might result from the selective pressure of RBD-specific neutralizing Abs (*55*).

The SARS-CoV-2 NTD undergoes rapid antigenic drift and accumulates a larger number of mutations and deletions relative to other regions of the S glycoprotein (*12*, *56*). For instance, the L18F substitution and the deletion of residue Y144 are found in 8% and 26% of viral genomes sequenced and are present in the B.1.351/P.1 lineages and the B.1.1.7 lineage, respectively. Both of these mutations are associated with reduction or abrogation of mAb binding and neutralization (*12*, *34*). The finding that multiple circulating SARS-CoV-2 variants map to the NTD, several of them in the antigenic supersite (site I), suggests that the NTD is subject to a strong selective pressure from the host humoral immune response. This is further supported by the identification of deletions within the NTD antigenic supersite in immunocompromised hosts with prolonged infections (*57*–*59*) and in the in vitro selection of SARS-CoV-2 S escape variants with NTD mutations that decrease the binding and neutralization potency of COVID-19 convalescent patient sera or mAbs (*12*, *34*, *60*, *61*). The data herein showing immune evasion of all tested NTD-specific mAbs by the B.1.427/B.1.429 variant also support that the NTD antigenic supersite is under host immune pressure.

Similar to how the S13I/W152C mutations facilitate evasion of all tested NTD-specific mAbs, E484K causes broad resistance to many RBD-specific mAbs. The independent acquisition of the E484K mutation in the B.1.351, P.1, and B.1.526 variants and, more recently, in the B.1.1.7 variant (*34*), suggests this could also occur in the B.1.427/B.1.429 lineages. Indeed, four genome sequences with the E484K RBD mutation in the B.1.427 variant have recently been deposited in GISAID. Alternatively, the S13I/W152C mutations could emerge in any of these variants. The S13I mutation was recently detected in the SARS-CoV-2 B.1.526 lineage, which was originally described in New York (*62*, *63*). Understanding the newly found mechanism of immune evasion in emerging SARS-CoV-2 variants, such as the signal peptide modification described herein, is as important as sequence surveillance itself to successfully counter the ongoing pandemic.

## Supplementary Material

20210701-1

## Supplementary Materials

science.sciencemag.org/content/373/6555/648/suppl/DC1
Materials and Methods Figs. S1 to S9 Tables S1 to S6 References (*64*–*90*) MDAR Reproducibility Checklist

## Appendix

View/request a protocol for this paper from *Bio-protocol*.
