# Supplementary material for: SARS-CoV-2 immune evasion by the B.1.427/B.1.429 variant of concern
Source: Science. 2021 Jul 1;373(6555):648–54. doi: 10.1126/science.abi7994 (PMC9835956; doi:10.1126/science.abi7994)
Supplement: 20210701-1 [file science.abi7994.v1.pdf]

Science

Cite as: McCallum *et al.*, *Science*
10.1126/science.abi7994 (2021).

SARS-CoV-2 immune evasion by the B.1.427/B.1.429 variant of concern

Matthew McCallum[1]†, Jessica Bassi[2]†, Anna De Marco[2]†, Alex Chen[3]†, Alexandra C. Walls[1]†, Julia Di Iulio[3], M. Alejandra Tortorici[1], Mary-Jane Navarro[1], Chiara Silacci-Fregni[2], Christian Saliba[2], Kaitlin R. Sprouse[1], Maria Agostini[3], Dora Pinto[2], Katja Culap[2], Siro Bianchi[2], Stefano Jaconi[2], Elisabetta Cameroni[2], John E. Bowen[1], Sasha W Tilles[4], Matteo Samuele Pizzuto[2], Sonja Bernasconi Guastalla[5], Giovanni Bona[6], Alessandra Franzetti Pellanda[6], Christian Garzoni[7], Wesley C. Van Voorhis[4], Laura E. Rosen[3], Gyorgy Snell[3], Amalio Telenti[3], Herbert W. Virgin[3], Luca Piccoli[2]*, Davide Corti[2]*, David Veesler[1]*

[1]Department of Biochemistry, University of Washington, Seattle, WA 98195, USA. [2]Humabs Biomed SA, a subsidiary of Vir Biotechnology, 6500 Bellinzona, Switzerland. [3]Vir Biotechnology, San Francisco, CA 94158, USA. [4]Center for Emerging and Re-emerging Infectious Diseases, Division of Allergy and Infectious Diseases, Department of Medicine, University of Washington School of Medicine, Seattle, WA 98195, USA. [5]Independent physician, 6828 Balerna, Switzerland. [6]Clinical Research Unit, Clinica Luganese Moncucco, 6900 Lugano, Switzerland. [7]Clinic of Internal Medicine and Infectious Diseases, Clinica Luganese Moncucco, 6900 Lugano, Switzerland.

†These authors contributed equally to this work.

*Corresponding author. Email: lpiccoli@vir.bio (L.P.); dcorti@vir.bio (D.C.); dveesler@uw.edu (D.V.)

A novel variant of concern (VOC) named CAL.20C (B.1.427/B.1.429), originally detected in California, carries spike glycoprotein mutations S13I in the signal peptide, W152C in the N-terminal domain (NTD), and L452R in the receptor-binding domain (RBD). Plasma from individuals vaccinated with a Wuhan-1 isolate-based mRNA vaccine or convalescent individuals exhibited neutralizing titers, which were reduced 2-3.5 fold against the B.1.427/B.1.429 variant relative to wildtype pseudoviruses. The L452R mutation reduced neutralizing activity of 14 out of 34 RBD-specific monoclonal antibodies (mAbs). The S13I and W152C mutations resulted in total loss of neutralization for 10 out of 10 NTD-specific mAbs since the NTD antigenic supersite was remodeled by a shift of the signal peptide cleavage site and formation of a new disulphide bond, as revealed by mass spectrometry and structural studies.

Coronavirus disease 2019 (COVID-19) is caused by SARS-CoV-2 and is associated with acute respiratory distress syndrome (ARDS), as well as extra-pulmonary complications such as vascular thrombosis, coagulopathy, and a hyperinflammatory syndrome contributing to disease severity and mortality. SARS-CoV-2 infects target cells using the spike glycoprotein (S) that is organized as a homotrimer with each monomer comprising an S_1 and an S_2 subunit (*1*, *2*). The S_1 subunit harbors the receptor-binding domain (RBD) and the N-terminal domain (NTD) as well as two other domains designated here as C and D (*3*, *4*). The RBD interacts with the angiotensin-converting enzyme 2 (ACE2) entry receptor on host cells through a subset of amino acids forming the receptor binding motif (RBM) (*1*, *2*, *5*–*7*). The NTD was suggested to bind DC-SIGN, L-SIGN, and AXL which may act as attachment receptors (*8*, *9*). Both the RBD and the NTD are targeted by neutralizing antibodies (Abs) in infected or vaccinated individuals and a subset of RBD-specific mAbs is currently being evaluated in clinical trials or are authorized for use in COVID-19 patients (*10*–*24*). The S_2 subunit is the fusion machinery that merges viral and host membranes to initiate infection and is the target of Abs cross-reacting with multiple coronavirus subgenera due to its higher sequence conservation compared to the S_1 subunit (*25*–*28*).

The ongoing global spread of SARS-CoV-2 led to the fixation of the D614G substitution (*29*, *30*) as well as to the emergence of a large number of viral lineages worldwide, including several variants of concern (VOC). Specifically, the B.1.1.7, B.1.351, and P.1 lineages that originated in the UK, South Africa, and Brazil, respectively, are characterized by the accumulation of S mutations as well as in other genes (*31*–*33*). Some of these mutations lead to significant reductions in the neutralization potency of several monoclonal Abs, convalescent sera and Pfizer/BioNTech BNT162b2- or Moderna mRNA-1273-elicited Abs (*19*, *34*–*40*). The B.1.1.7 variant has become dominant worldwide due to its higher transmissibility (*33*), underscoring the importance of studying and understanding the consequences of SARS-CoV-2 antigenic drift.

Results

The incidence of the B.1.427/B.1.429 lineages is increasing rapidly

The SARS-CoV-2 B.1.427/B.1.429 variant was reported for the first time at the beginning of 2021 in California and as of May 2021 has been detected in 34 additional countries (*41*, *42*). The two lineages B.1.427 and B.1.429 (belonging to clade 20C according to Nextstrain designation) share the same S

mutations (S13I, and W152C in the NTD and L452R in the RBD), but harbor different mutations in other SARS-CoV-2 genes (*42*). Molecular clock analysis suggest that the progenitor of both lineages emerged in May 2020, diverging to give rise to the B.1.427 and B.1.429 lineages in June-July 2020 (*42*). The fast rise in the number of cases associated with the B.1.427/B.1.429 lineages led to their classification as a VOC by the US Center for Disease Control (https://www.cdc.gov/coronavirus/2019-ncov/cases-updates/variant-surveillance/variant-info.html).

As of April 30, 2021, 8,441 and 21,072 sequenced genomes are reported in GISAID for the B.1.427 and B.1.429 lineages, respectively. This VOC was detected in California and in other US states, and more recently in 34 additional countries worldwide (Fig. 1, A to H, and table S1). The number of B.1.427/B.1.429 genome sequences deposited increased rapidly after December 2020, with an incidence exceeding 50% in California since February 2021 (Fig. 1, B to E). Collectively, this analysis illustrates the increased incidence of the B.1.427/B.1.429 VOC, and its progressive geographical spread from California to other US states and other countries, which is consistent with a recent study suggesting enhanced transmissibility relative to the ancestral isolate (*42*).

B.1.427/ B.1.429 S reduces sensitivity to vaccine-elicited Abs

To assess the impact of the three mutations present in the B.1.427/B.1.429 S glycoprotein on neutralization, we first compared side-by-side the neutralization potency of mRNA vaccine-elicited Abs against G614 S and B.1.427/B.1.429 S pseudoviruses. We used plasma from fifteen individuals who received two doses of Moderna mRNA-1273 vaccine and from fifteen individuals who received two doses of Pfizer/BioNtech BNT162b2 vaccine collected between 7 and 27 days after booster immunization (table S2). All vaccinees had substantial plasma neutralizing activity against G614 SARS-CoV-2 S pseudotyped viruses. Using a murine leukemia virus (MLV) pseudotyping system, geometric mean titers (GMTs) showed that the average neutralization potency of the Moderna mRNA1273-elicited plasma was reduced 2.4-fold for B.1.427/B.1.429 S (GMT: 178) compared to G614 S (GMT: 424) (Fig. 2, A and B; figs. S1 and S2; and table S3) whereas it was reduced 2.3-fold with Pfizer/BioNtech BNT162b2-elicited plasma (B.1.427/B.1.429 GMT: 78 versus G614 GMT: 182) (Fig. 2, C and D; figs. S1 and S2; and table S3). Using a vesicular stomatitis virus (VSV) pseudotyping system, we observed a 2.2-fold average reduction of Moderna mRNA1273-elicited plasma neutralizing activity against B.1.427/B.1.429 S (GMT: 213) compared to G614 S (GMT: 464) pseudoviruses (Fig. 2, E and F; figs. S1 and S2; and table S3) and a 2.5-fold average reduction of Pfizer/BioNtech BNT162b2-elicited plasma neutralizing activity against B.1.427/B.1.429 S (GMT: 113) compared to G614 S (GMT: 285) pseudoviruses (Fig. 2, G and H; figs. S1 and S2; and table S3). We also analyzed plasma from 18 individuals, 5 of whom were previously infected with wildtype SARS-CoV-2, who received two doses of Pfizer/BioNtech BNT162b2 vaccine and whose samples were collected between 14 and 28 days after booster immunization. We compared the neutralization potency of Pfizer/BioNtech BNT162b2 vaccine-elicited Abs against D614 S, B.1.427/B.1.429 S, B.1.1.7 S, B.1.351 S and P.1 S VSV pseudotyped viruses using Vero E6 expressing TMPRSS2 as target cells. GMTs plasma neutralization potency was reduced 2.9-fold for B.1.427/B.1.429 S (GMT: 197) compared to D614 S (GMT: 570), which is a comparable decrease to that observed with B.1.351 (GMT: 180, 3.2-fold reduction) and greater to that observed with B.1.1.7 and P.1 (GMT: 450 and 330, 1.3-fold and 1.7-fold reduction, respectively) pseudotyped viruses (Fig. 2, I and J; figs. S1 and S2; and table S3). These data indicate that the three B.1.427/B.1.429 S residue substitutions lead to a modest but significant reduction of neutralization potency from vaccine-elicited Abs.

We also analyzed plasma from 9 convalescent donors, who experienced symptomatic COVID-19 in early 2020 (and consequently were likely exposed to the Wuhan-1 or a closely related SARS-CoV-2 isolate) collected 15 to 28 days post symptom onset (table S2). The neutralization potency of the 9 convalescent donor plasma was reduced 3.4-fold for B.1.427/B.1.429 S (GMT: 70) compared to G614 S (GMT: 240), similar to what we observed with B.1.351 (4.4-fold, GMT: 55) and P.1 (3.3-fold, GMT: 72) pseudotyped viruses, whereas neutralization of B.1.1.7 was less affected (1.9-fold, GMT: 127) (Fig. 2, K and L; figs. S1 and S2; and table S3). In several cases the level of neutralizing activity against the VOC was found to be below the limit of detection.

These findings show that the three mutations present in the B1.427/B.1.429 S glycoprotein decrease the neutralizing activity of vaccine-elicited and infection-elicited Abs, suggesting that these lineage-defining residue substitutions are associated with immune evasion. However, these data also underscore the higher quality of Ab responses induced by vaccination compared to infection and their enhanced resilience to mutations found in VOC.

B.1.427/B.1.429 S mutations reduce sensitivity to RBD- and NTD-specific Abs

To evaluate the contribution of RBD and NTD substitutions to the reduced neutralization potency of sera from vaccinees and convalescent plasma, we compared the neutralizing activity of 34 RBD and 10 NTD mAbs against the D614 S or B.1.427/B.1.429 S variant using a VSV pseudotyping system (*1*, *43*).

The panel of RBD-specific mAbs (including 6 clinical mAbs) recognize distinct antigenic sites as previously

characterized (*10*, *11*, *13*, *20*, *44*, *45*). Briefly, epitopes span the RBM (antigenic sites Ia and Ib), a cryptic antigenic site II, the exposed N343 glycan-containing antigenic site IV and a second cryptic antigenic site V (*10*, *11*). A total of 14 out of 34 mAbs showed a reduced neutralization potency when comparing B.1.427/B.1.429 S and D614 S pseudoviruses (Fig. 3, A to C, and fig. S3). Considering the 6 mAbs in clinical use: regdanvimab (CT-P59), and to a smaller extent etesevimab (LY-CoV016), showed a reduction in neutralization potency, whereas bamlanivimab (LY-CoV555) entirely lost its neutralizing activity. Neutralization mediated by the casirivimab/imdevimab mAb cocktail (REGN10933 and REGN10987) (*14*, *15*), and by VIR-7831 (derivative of S309, recently renamed sotrovimab) (*10*, *23*, *24*), is unaffected by the B.1.427/B.1.429 S variant. To address the role of the L452R mutation in the neutralization escape from RBD-specific Abs, we tested the binding of the 34 RBD-specific mAbs to WT and L452R mutant RBD by biolayer interferometry (fig. S4). The 10 RBD-specific mAbs experiencing a 10-fold or greater reduction in neutralization potency of the B.1.427/B.1.429 variant, relative to D614 S, bound poorly to the L452R RBD mutant, demonstrating a direct role of this mutation im immune evasion.

We found that the neutralizing activity of all 10 NTD-specific mAbs tested was abolished as a result of the presence of the S13I and W152C mutations (Fig. 3, D to F). These data indicate that the decreased potency of neutralization of the B.1.427/B.1.429 variant results from evasion of both RBD- and NTD-specific mAb-mediated neutralization.

Structural characterization of the SARS-CoV-2 B.1.427/B.1.429 S trimer

To visualize the changes in SARS-CoV-2 B.1.427/B.1.429 S that contribute to immune evasion, we determined a cryoEM structure of the variant S ectodomain trimer (carrying the HexaPro mutations (*46*)) bound to the RBD-specific mAb S2M11 and the NTD-specific mAb S2L20 at 2.3 Å resolution (Fig. 4A, fig. S5, and table S4). S2M11 was used to lock the RBDs in the closed state (Fig. 4B) whereas S2L20 was used to stabilize the NTDs (Fig. 4C) (*12*, *13*). Superimposing the regdanvimab- (CT-P59) and bamlanivimab- (LY-CoV555) bound SARS-CoV-2 RBD structures to B.1.427/B.1.429 S reveal that the introduced L452R is sterically incompatible with binding of these mAbs (Fig. 4, D and E), rationalizing the dampening or loss of neutralizing activity.

We subsequently used local refinement to account for the conformational dynamics of the NTD and S2L20 relative to the rest of S and obtained a cryoEM reconstruction of the NTD bound to S2L20 at 3.0 Å resolution (Fig. 4C, fig. S5, and table S4). The structure reveals that the B.1.427/B.1.429 NTD antigenic supersite is severely altered. The N terminus is disordered up to residue 27, as is the supersite β-hairpin (disordered between residues 137-158) and the supersite loop (disordered between residues 243-264) (Fig. 4F). These structural changes explain the abrogation of binding and neutralization of the panel of NTD-specific mAbs evaluated.

Overlaying an ACE2-bound SARS-CoV-2 RBD structure with the B.1.427/B.1.429 variant S structure shows that the R452 residue points away from and does not contact ACE2, suggesting that this substitution would not affect receptor engagement (Fig. 4G). We next evaluated binding of the monomeric human ACE2 ectodomain to immobilized B.1.427/B.1.429 and wildtype RBDs using surface plasmon resonance (fig. S6, A and B, and table S5) biolayer interferometry (fig. S6, C to E, and table S5) as well as binding of B.1.427/B.1.429, B.1.1.7 and wildtype RBDs to immobilized human ACE2 by ELISA (fig. S6F and table SS5). Our results indicate that the B.1.427/B.1.429 and wildtype RBDs bound to ACE2 with comparable affinities (whereas the B.1.1.7 RBD had a markedly increased affinity for ACE2 (*34*)), validating the structural observations.

Disulfide bond rearrangement in the B.1.427/B.1.429 variant NTD antigenic supersite

To investigate further the molecular basis for the loss of NTD-directed mAb neutralizing activity and structural changes in the NTD, we analyzed binding of a panel of NTD-specific mAbs to recombinant SARS-CoV-2 NTD variants using ELISA. The S13I signal peptide mutation dampened binding of 5 mAbs and abrogated binding of 5 additional mAbs out of 11 neutralizing mAbs evaluated (Fig. 5A and fig. S7). Furthermore, the W152C mutation reduced recognition of 6 NTD neutralizing mAbs, including a complete loss of binding for two of them, with a complementary pattern to that observed for S13I (Fig. 5A and fig. S7). The B.1.427/B.1.429 S13I/W152C NTD did not bind to any NTD-directed neutralizing mAbs, which are known to target a single antigenic site (antigenic site i) (*12*), whereas binding of the non-neutralizing S2L20 mAb to the NTD antigenic site iv was not affected by any mutants, confirming proper retention of folding, as supported by the structural data (Fig. 5A and fig. S7). Binding of vaccine-elicited plasma to NTD mutants confirmed and extended these observations with polyclonal Abs by showing an increasingly marked reduction in binding titers due to the W152C, S13I and S13I/W152C residue substitutions (Fig. 5B and fig. S8).

We previously showed that disruption of the C15/C136 disulfide bond that connects the N terminus to the rest of the NTD, through mutation of either residue or alteration of the signal peptide cleavage site, abrogates the neutralizing activity of mAbs targeting the NTD antigenic supersite (site i) (*12*). As the S13I substitution resides in the signal peptide and is predicted to shift the signal peptide cleavage site from S13-Q14 to C15-V16, we hypothesized that this substitution indirectly affects the integrity of NTD antigenic site i, which

comprises the N terminus. Mass spectrometry analysis of the S13I and S13I/W152C NTD variants confirmed that signal peptide cleavage occurs immediately after residue C15 (Fig. 5, C to E). As a result, C136, which would otherwise be disulfide linked to C15, is cysteinylated in the S13I NTD due to the presence of free cysteine in the expression media (Fig. 5D and fig. S9). Likewise, the W152C mutation, which introduces a free cysteine, was also found to be cysteinylated in the W152C NTD (Fig. 5E). It is not clear if cysteinylation would occur during natural infection with S13I or W152C mutants alone, or what contribution cysteinylation plays in immune evasion of S13I or W152C mutants alone. Notably, dampening of NTD-specific neutralizing mAb binding is stronger for the S13I mutant than for the S12P mutant which we previously showed to also shifts the signal peptide cleavage site to C15-V16 (Fig. 5A). Conversely, we did not observe any effect on mAb binding of the S12F substitution, which has also been detected in clinical isolates, in agreement with the fact that this mutation did not affect the native signal peptide cleavage site (i.e., it occurs at the S13-Q14 position), as observed by mass spectrometry (Fig. 5G). In the absence of the C15-C136 disulfide bond the N terminus is no longer stapled to the NTD, consistent with the structural data showing that the N terminus of the B.1.427/B.1.429 variant becomes disordered relative to the rest of the NTD (Fig. 4C).

Although the S13I and W152C NTD variants were respectively cysteinylated at positions C136 and W152C, the double mutant S13I/W152C was not cysteinylated, suggesting that C136 and W152C had formed a new disulfide bond. (Fig. 5, D to F). Tandem mass-spectrometry analysis of non-reduced, digested peptides identified linked discontinuous peptides containing C136 and W152C (fig. S9) confirming that a disulfide bond forms between C136 and W152C in the S13I/W152C NTD of the B.1.427/B.1.429 variant. W152C is in the β-hairpin of the antigenic supersite, and the formation of a new disulfide bond with C136 would move residues in the β-hairpin >20 Å and the local structure of the β-hairpin was disordered in the B.1.427/B.1.429 variant (Fig. 4C).

Collectively, these findings demonstrate that the S13I and W152C mutations found in the B.1.427/B.1.429 S variant are jointly responsible for escape from NTD-specific mAbs, due to deletion of the SARS-CoV-2 S two N-terminal residues and overall rearrangement of the NTD antigenic supersite. Our data support that the SARS-CoV-2 NTD evolved a compensatory mechanism to form an alternative disulfide bond and that mutations of the S signal peptide occur in vivo in a clinical setting to promote immune evasion. The SARS-CoV-2 B.1.427/B.1.429 S variant therefore relies on an indirect and unusual neutralization-escape strategy.

Discussion

Serum or plasma neutralizing activity is a correlate of protection against SARS-CoV-2 challenge in non-human primates (*47*, *48*) and treatment with several neutralizing mAbs has reduced viral burden and decreased hospitalization and mortality in clinical trials (*10*, *14*, *15*, *22*, *23*, *49*). The observed L452R-mediated immune evasion of B.1.427/B.1.429 S concurs with previous findings that this substitution reduced the binding or neutralizing activity of some mAbs prior to the description of the B.1.427/B.1.429 variant (*50*–*53*). The acquisition of the L452R substitution by multiple lineages across multiple continents, including the B.1.617.1 and B.1.617.2 lineages emerging in India (*54*), is suggestive of positive selection, which might result from the selective pressure of RBD-specific neutralizing Abs (*55*).

The SARS-CoV-2 NTD undergoes rapid antigenic drift and accumulates a larger number of mutations and deletions relative to other regions of the S glycoprotein (*12*, *56*). For instance, the L18F substitution and the deletion of residue Y144 are found in 8% and 26% of viral genomes sequenced and are present in the B.1.351/P.1 lineages and the B.1.1.7 lineage, respectively. Both of these mutations are associated with reduction or abrogation of mAb binding and neutralization (*12*, *34*). The finding that multiple circulating SARS-CoV-2 variants map to the NTD, including several of them in the antigenic supersite (site i), suggests that the NTD is subject to a strong selective pressure from the host humoral immune response. This is further supported by the identification of deletions within the NTD antigenic supersite in immunocompromised hosts with prolonged infections (*57*–*59*) and the in vitro selection of SARS-CoV-2 S escape variants with NTD mutations that decrease binding and neutralization potency of COVID-19 convalescent patient sera or mAbs (*12*, *34*, *60*, *61*). The data herein showing immune evasion of all tested NTD-specific mAbs by the B.1.427/B.1.429 variant also support that the NTD antigenic supersite is under host immune pressure.

Similar to how the S13I/W152C mutations facilitate evasion of all tested NTD-specific mAbs, E484K causes broad resistance to many RBD-specific mAbs. The independent acquisition of the E484K mutation in the B.1.351, P.1, B.1.526 variants and more recently the B.1.1.7 variant (*34*) suggests this could also occur in the B.1.427/B.1.429 lineages. Indeed, 4 genome sequences with the E484K RBD mutation in the B.1.427 variant have recently been deposited in GISAID. Alternatively, the S13I/W152C mutations could emerge in any of these variants. We note that the S13I mutation was recently detected in the SARS-CoV-2 B.1.526 lineage, which was originally described in New York (*62*, *63*). Understanding the newfound mechanism of immune evasion of the emerging variants, such as the signal peptide modification described herein, is as important as sequence surveillance itself to successfully counter the ongoing pandemic.

REFERENCES AND NOTES

1. A. C. Walls, Y. J. Park, M. A. Tortorici, A. Wall, A. T. McGuire, D. Veesler, Structure, function, and antigenicity of the SARS-CoV-2 spike glycoprotein. *Cell* **181**, 281–292.e6 (2020). doi:10.1016/j.cell.2020.02.058 Medline
2. D. Wrapp, N. Wang, K. S. Corbett, J. A. Goldsmith, C. L. Hsieh, O. Abiona, B. S. Graham, J. S. McLellan, Cryo-EM structure of the 2019-nCoV spike in the prefusion conformation. *Science* **367**, 1260–1263 (2020). doi:10.1126/science.abb2507 Medline

3. A. C. Walls, M. A. Tortorici, B. J. Bosch, B. Frenz, P. J. M. Rottier, F. DiMaio, F. A. Rey, D. Veesler, Cryo-electron microscopy structure of a coronavirus spike glycoprotein trimer. *Nature* **531**, 114–117 (2016). doi:10.1038/nature16988 Medline
4. M. A. Tortorici, D. Veesler, Structural insights into coronavirus entry. *Adv. Virus Res.* **105**, 93–116 (2019). doi:10.1016/bs.aivir.2019.08.002 Medline
5. M. Letko, A. Marzi, V. Munster, Functional assessment of cell entry and receptor usage for SARS-CoV-2 and other lineage B betacoronaviruses. *Nat. Microbiol.* **5**, 562–569 (2020). doi:10.1038/s41564-020-0688-y Medline
6. P. Zhou, X. L. Yang, X. G. Wang, B. Hu, L. Zhang, W. Zhang, H. R. Si, Y. Zhu, B. Li, C. L. Huang, H. D. Chen, J. Chen, Y. Luo, H. Guo, R. D. Jiang, M. Q. Liu, Y. Chen, X. R. Shen, X. Wang, X. S. Zheng, K. Zhao, Q. J. Chen, F. Deng, L. L. Liu, B. Yan, F. X. Zhan, Y. Y. Wang, G. F. Xiao, Z. L. Shi, Addendum: A pneumonia outbreak associated with a new coronavirus of probable bat origin. *Nature* **588**, E6 (2020). doi:10.1038/s41586-020-2951-z Medline
7. M. Hoffmann, H. Kleine-Weber, S. Schroeder, N. Krüger, T. Herrler, S. Erichsen, T. S. Schiergens, G. Herrler, N. H. Wu, A. Nitsche, M. A. Müller, C. Drosten, S. Pöhlmann, SARS-CoV-2 cell entry depends on ACE2 and TMPRSS2 and is blocked by a clinically proven protease inhibitor. *Cell* **181**, 271–280.e8 (2020). doi:10.1016/j.cell.2020.02.052 Medline
8. W. T. Soh, Y. Liu, E. E. Nakayama, C. Ono, S. Torii, H. Nakagami, Y. Matsuura, T. Shioda, H. Arase, The N-terminal domain of spike glycoprotein mediates SARS-CoV-2 infection by associating with L-SIGN and DC-SIGN. bioRxiv 369264 [Preprint]. 5 November 2020. https://doi.org/10.1101/2020.11.05.369264.
9. S. Wang, Z. Qiu, Y. Hou, X. Deng, W. Xu, T. Zheng, P. Wu, S. Xie, W. Bian, C. Zhang, Z. Sun, K. Liu, C. Shan, A. Lin, S. Jiang, Y. Xie, Q. Zhou, L. Lu, J. Huang, X. Li, AXL is a candidate receptor for SARS-CoV-2 that promotes infection of pulmonary and bronchial epithelial cells. *Cell Res.* **31**, 126–140 (2021). doi:10.1038/s41422-020-00460-y Medline
10. D. Pinto, Y. J. Park, M. Beltramello, A. C. Walls, M. A. Tortorici, S. Bianchi, S. Jaconi, K. Culap, F. Zatta, A. De Marco, A. Peter, B. Guarino, R. Spreafico, E. Cameroni, J. B. Case, R. E. Chen, C. Havenar-Daughton, G. Snell, A. Telenti, H. W. Virgin, A. Lanzavecchia, M. S. Diamond, K. Fink, D. Veesler, D. Corti, Cross-neutralization of SARS-CoV-2 by a human monoclonal SARS-CoV antibody. *Nature* **583**, 290–295 (2020). doi:10.1038/s41586-020-2349-y Medline
11. L. Piccoli, Y. J. Park, M. A. Tortorici, N. Czudnochowski, A. C. Walls, M. Beltramello, C. Silacci-Fregni, D. Pinto, L. E. Rosen, J. E. Bowen, O. J. Acton, S. Jaconi, B. Guarino, A. Minola, F. Zatta, N. Sprugasci, J. Bassi, A. Peter, A. De Marco, J. C. Nix, F. Mele, S. Jovic, B. F. Rodriguez, S. V. Gupta, F. Jin, G. Piumatti, G. Lo Presti, A. F. Pellanda, M. Biggiogero, M. Tarkowski, M. S. Pizzuto, E. Cameroni, C. Havenar-Daughton, M. Smithey, D. Hong, V. Lepori, E. Albanese, A. Ceschi, E. Bernasconi, L. Elzi, P. Ferrari, C. Garzoni, A. Riva, G. Snell, F. Sallusto, K. Fink, H. W. Virgin, A. Lanzavecchia, D. Corti, D. Veesler, Mapping neutralizing and immunodominant sites on the SARS-CoV-2 spike receptor-binding domain by structure-guided high-resolution serology. *Cell* **183**, 1024–1042.e21 (2020). doi:10.1016/j.cell.2020.09.037 Medline
12. M. McCallum, A. De Marco, F. A. Lempp, M. A. Tortorici, D. Pinto, A. C. Walls, M. Beltramello, A. Chen, Z. Liu, F. Zatta, S. Zepeda, J. di Iulio, J. E. Bowen, M. Montiel-Ruiz, J. Zhou, L. E. Rosen, S. Bianchi, B. Guarino, C. S. Fregni, R. Abdelnabi, S. C. Foo, P. W. Rothlauf, L.-M. Bloyet, F. Benigni, E. Cameroni, J. Neyts, A. Riva, G. Snell, A. Telenti, S. P. J. Whelan, H. W. Virgin, D. Corti, M. S. Pizzuto, D. Veesler, N-terminal domain antigenic mapping reveals a site of vulnerability for SARS-CoV-2. *Cell* **184**, 2332–2347.e16 (2021). doi:10.1016/j.cell.2021.03.028 Medline
13. M. A. Tortorici, M. Beltramello, F. A. Lempp, D. Pinto, H. V. Dang, L. E. Rosen, M. McCallum, J. Bowen, A. Minola, S. Jaconi, F. Zatta, A. De Marco, B. Guarino, S. Bianchi, E. J. Lauron, H. Tucker, J. Zhou, A. Peter, C. Havenar-Daughton, J. A. Wojcechowskyj, J. B. Case, R. E. Chen, H. Kaiser, M. Montiel-Ruiz, M. Meury, N. Czudnochowski, R. Spreafico, J. Dillen, C. Ng, N. Sprugasci, K. Culap, F. Benigni, R. Abdelnabi, S. C. Foo, M. A. Schmid, E. Cameroni, A. Riva, A. Gabrieli, M. Galli, M. S. Pizzuto, J. Neyts, M. S. Diamond, H. W. Virgin, G. Snell, D. Corti, K. Fink, D. Veesler, Ultrapotent human antibodies protect against SARS-CoV-2 challenge via multiple mechanisms. *Science* **370**, 950–957 (2020). doi:10.1126/science.abe3354 Medline
14. J. Hansen, A. Baum, K. E. Pascal, V. Russo, S. Giordano, E. Wloga, B. O. Fulton, Y. Yan, K. Koon, K. Patel, K. M. Chung, A. Hermann, E. Ullman, J. Cruz, A. Rafique, T. Huang, J. Fairhurst, C. Libertiny, M. Malbec, W. Y. Lee, R. Welsh, G. Farr, S. Pennington, D. Deshpande, J. Cheng, A. Watty, P. Bouffard, R. Babb, N. Levenkova, C. Chen, B. Zhang, A. Romero Hernandez, K. Saotome, Y. Zhou, M. Franklin, S. Sivapalasingam, D. C. Lye, S. Weston, J. Logue, R. Haupt, M. Frieman, G. Chen, W. Olson, A. J. Murphy, N. Stahl, G. D. Yancopoulos, C. A. Kyratsous, Studies in humanized mice and convalescent humans yield a SARS-CoV-2 antibody cocktail. *Science* **369**, 1010–1014 (2020). doi:10.1126/science.abd0827 Medline
15. A. Baum, B. O. Fulton, E. Wloga, R. Copin, K. E. Pascal, V. Russo, S. Giordano, K. Lanza, N. Negron, M. Ni, Y. Wei, G. S. Atwal, A. J. Murphy, N. Stahl, G. D. Yancopoulos, C. A. Kyratsous, Antibody cocktail to SARS-CoV-2 spike protein prevents rapid mutational escape seen with individual antibodies. *Science* **369**, 1014–1018 (2020). doi:10.1126/science.abd0831 Medline
16. N. Suryadevara, S. Shrihari, P. Gilchuk, L. A. VanBlargan, E. Binshtein, S. J. Zost, R. S. Nargi, R. E. Sutton, E. S. Winkler, E. C. Chen, M. E. Fouch, E. Davidson, B. J. Doranz, R. E. Chen, P.-Y. Shi, R. H. Carnahan, L. B. Thackray, M. S. Diamond, J. E. Crowe Jr., Neutralizing and protective human monoclonal antibodies recognizing the N-terminal domain of the SARS-CoV-2 spike protein. *Cell* **184**, 2316–2331.e15 (2021). doi:10.1016/j.cell.2021.03.029 Medline
17. G. Cerutti, Y. Guo, T. Zhou, J. Gorman, M. Lee, M. Rapp, E. R. Reddem, J. Yu, F. Bahna, J. Bimela, Y. Huang, P. S. Katsamba, L. Liu, M. S. Nair, R. Rawi, A. S. Olia, P. Wang, B. Zhang, G.-Y. Chuang, D. D. Ho, Z. Sheng, P. D. Kwong, L. Shapiro, Potent SARS-CoV-2 neutralizing antibodies directed against spike N-terminal domain target a single supersite. *Cell Host Microbe* **29**, 819–833.e7 (2021). doi:10.1016/j.chom.2021.03.005 Medline
18. X. Chi, R. Yan, J. Zhang, G. Zhang, Y. Zhang, M. Hao, Z. Zhang, P. Fan, Y. Dong, Y. Yang, Z. Chen, Y. Guo, J. Zhang, Y. Li, X. Song, Y. Chen, L. Xia, L. Fu, L. Hou, J. Xu, C. Yu, J. Li, Q. Zhou, W. Chen, A neutralizing human antibody binds to the N-terminal domain of the Spike protein of SARS-CoV-2. *Science* **369**, 650–655 (2020). doi:10.1126/science.abc6952 Medline
19. Z. Wang, F. Schmidt, Y. Weisblum, F. Muecksch, C. O. Barnes, S. Finkin, D. Schaefer-Babajew, M. Cipolla, C. Gaebler, J. A. Lieberman, T. Y. Oliveira, Z. Yang, M. E. Abernathy, K. E. Huey-Tubman, A. Hurley, M. Turroja, K. A. West, K. Gordon, K. G. Millard, V. Ramos, J. Da Silva, J. Xu, R. A. Colbert, R. Patel, J. Dizon, C. Unson-O'Brien, I. Shimeliovich, A. Gazumyan, M. Caskey, P. J. Bjorkman, R. Casellas, T. Hatziioannou, P. D. Bieniasz, M. C. Nussenzweig, mRNA vaccine-elicited antibodies to SARS-CoV-2 and circulating variants. *Nature* **592**, 616–622 (2021). doi:10.1038/s41586-021-03324-6 Medline
20. C. O. Barnes, C. A. Jette, M. E. Abernathy, K. A. Dam, S. R. Esswein, H. B. Gristick, A. G. Malyutin, N. G. Sharaf, K. E. Huey-Tubman, Y. E. Lee, D. F. Robbiani, M. C. Nussenzweig, A. P. West Jr., P. J. Bjorkman, SARS-CoV-2 neutralizing antibody structures inform therapeutic strategies. *Nature* **588**, 682–687 (2020). doi:10.1038/s41586-020-2852-1 Medline
21. D. F. Robbiani, C. Gaebler, F. Muecksch, J. C. C. Lorenzi, Z. Wang, A. Cho, M. Agudelo, C. O. Barnes, A. Gazumyan, S. Finkin, T. Hägglöf, T. Y. Oliveira, C. Viant, A. Hurley, H. H. Hoffmann, K. G. Millard, R. G. Kost, M. Cipolla, K. Gordon, F. Bianchini, S. T. Chen, V. Ramos, R. Patel, J. Dizon, I. Shimeliovich, P. Mendoza, H. Hartweger, L. Nogueira, M. Pack, J. Horowitz, F. Schmidt, Y. Weisblum, E. Michailidis, A. W. Ashbrook, E. Waltari, J. E. Pak, K. E. Huey-Tubman, N. Koranda, P. R. Hoffman, A. P. West Jr., C. M. Rice, T. Hatziioannou, P. J. Bjorkman, P. D. Bieniasz, M. Caskey, M. C. Nussenzweig, Convergent antibody responses to SARS-CoV-2 in convalescent individuals. *Nature* **584**, 437–442 (2020). doi:10.1038/s41586-020-2456-9 Medline
22. B. E. Jones, P. L. Brown-Augsburger, K. S. Corbett, K. Westendorf, J. Davies, T. P. Cujec, C. M. Wiethoff, J. L. Blackbourne, B. A. Heinz, D. Foster, R. E. Higgs, D. Balasubramaniam, L. Wang, R. Bidshahri, L. Kraft, Y. Hwang, S. Žentelis, K. R. Jepson, R. Goya, M. A. Smith, D. W. Collins, S. J. Hinshaw, S. A. Tycho, D. Pellacani, P. Xiang, K. Muthuraman, S. Sobhanifar, M. H. Piper, F. J. Triana, J. Hendle, A. Pustilnik, A. C. Adams, S. J. Berens, R. S. Baric, D. R. Martinez, R. W. Cross, T. W. Geisbert, V. Borisevich, O. Abiona, H. M. Belli, M. de Vries, A. Mohamed, M. Dittmann, M. Samanovic, M. J. Mulligan, J. A. Goldsmith, C. L. Hsieh, N. V. Johnson, D. Wrapp, J. S. McLellan, B. C. Barnhart, B. S. Graham, J. R. Mascola, C. L. Hansen, E. Falconer, The neutralizing antibody, LY-CoV555, protects against SARS-CoV-2 infection in nonhuman primates. *Sci. Transl. Med.* **13**, eabf1906 (2020). doi:10.1126/scitranslmed.abf1906 Medline

23. A. L. Cathcart, C. Havenar-Daughton, F. A. Lempp, D. Ma, M. Schmid, M. L. Agostini, B. Guarino, J. Diiulio, L. Rosen, H. Tucker, J. Dillen, S. Subramanian, B. Sloan, S. Bianchi, J. Wojcechowskyj, J. Zhou, H. Kaiser, A. Chase, M. Montiel-Ruiz, N. Czudnochowski, E. Cameroni, S. Ledoux, C. Colas, L. Soriaga, A. Telenti, S. Hwang, G. Snell, H. W. Virgin, D. Corti, C. M. Hebner, The dual function monoclonal antibodies VIR-7831 and VIR-7832 demonstrate potent in vitro and in vivo activity against SARS-CoV-2. bioRxiv 434607 [Preprint]. 24 June 2021. https://doi.org/10.1101/2021.03.09.434607
24. D. Corti, L. A. Purcell, G. Snell, D. Veesler, Tackling COVID-19 with neutralizing monoclonal antibodies. *Cell* **184**, 3086–3108 (2021). doi:10.1016/j.cell.2021.05.005 Medline
25. C. Wang, R. van Haperen, J. Gutiérrez-Álvarez, W. Li, N. M. A. Okba, I. Albulescu, I. Widjaja, B. van Dieren, R. Fernandez-Delgado, I. Sola, D. L. Hurdiss, O. Daramola, F. Grosveld, F. J. M. van Kuppeveld, B. L. Haagmans, L. Enjuanes, D. Drabek, B.-J. Bosch, A conserved immunogenic and vulnerable site on the coronavirus spike protein delineated by cross-reactive monoclonal antibodies. *Nat. Commun.* **12**, 1715 (2021). doi:10.1038/s41467-021-21968-w Medline
26. G. Song, W. T. He, S. Callaghan, F. Anzanello, D. Huang, J. Ricketts, J. L. Torres, N. Beutler, L. Peng, S. Vargas, J. Cassell, M. Parren, L. Yang, C. Ignacio, D. M. Smith, J. E. Voss, D. Nemazee, A. B. Ward, T. Rogers, D. R. Burton, R. Andrabi, Cross-reactive serum and memory B-cell responses to spike protein in SARS-CoV-2 and endemic coronavirus infection. *Nat. Commun.* **12**, 2938 (2021). doi:10.1038/s41467-021-23074-3 Medline
27. D. Pinto, M. M. Sauer, N. Czudnochowski, J. S. Low, M. Alejandra Tortorici, M. P. Housley, J. Noack, A. C. Walls, J. E. Bowen, B. Guarino, L. E. Rosen, J. di Iulio, J. Jerak, H. Kaiser, S. Islam, S. Jaconi, N. Sprugasci, K. Culap, R. Abdelnabi, C. S.-Y. Foo, L. Coelmont, I. Bartha, S. Bianchi, C. Silacci-Fregni, J. Bassi, R. Marzi, E. Vetti, A. Cassotta, A. Ceschi, P. Ferrari, P. E. Cippa', O. Giannini, S. Ceruti, A. Riva, F. Benigni, E. Cameroni, L. Piccoli, M. S. Pizzuto, M. Smithey, D. Hong, A. Telenti, F. A. Lempp, J. Neyts, C. Havenar-Daughton, A. Lanzavecchia, F. Sallusto, G. Snell, H. W. Virgin, M. Beltramello, D. Corti, D. Veesler, A human antibody that broadly neutralizes betacoronaviruses protects against SARS-CoV-2 by blocking the fusion machinery. bioRxiv 442808 [Preprint]. 10 May 2021. https://doi.org/10.1101/2021.05.09.442808.
28. M. M. Sauer, M. A. Tortorici, Y.-J. Park, A. C. Walls, L. Homad, O. J. Acton, J. E. Bowen, C. Wang, X. Xiong, W. de van der Schueren, J. Quispe, B. G. Hoffstrom, B. J. Bosch, A. T. McGuire, D. Veesler, Structural basis for broad coronavirus neutralization. *Nat. Struct. Mol. Biol.* **28**, 478–486 (2021). doi:10.1038/s41594-021-00596-4 Medline
29. B. Korber, W. M. Fischer, S. Gnanakaran, H. Yoon, J. Theiler, W. Abfalterer, N. Hengartner, E. E. Giorgi, T. Bhattacharya, B. Foley, K. M. Hastie, M. D. Parker, D. G. Partridge, C. M. Evans, T. M. Freeman, T. I. de Silva, C. McDanal, L. G. Perez, H. Tang, A. Moon-Walker, S. P. Whelan, C. C. LaBranche, E. O. Saphire, D. C. Montefiori, A. Angyal, R. L. Brown, L. Carrilero, L. R. Green, D. C. Groves, K. J. Johnson, A. J. Keeley, B. B. Lindsey, P. J. Parsons, M. Raza, S. Rowland-Jones, N. Smith, R. M. Tucker, D. Wang, M. D. Wyles; Sheffield COVID-19 Genomics Group, Tracking changes in SARS-CoV-2 spike: Evidence that D614G increases infectivity of the COVID-19 virus. *Cell* **182**, 812–827.e19 (2020). doi:10.1016/j.cell.2020.06.043 Medline
30. J. A. Plante, Y. Liu, J. Liu, H. Xia, B. A. Johnson, K. G. Lokugamage, X. Zhang, A. E. Muruato, J. Zou, C. R. Fontes-Garfias, D. Mirchandani, D. Scharton, J. P. Bilello, Z. Ku, Z. An, B. Kalveram, A. N. Freiberg, V. D. Menachery, X. Xie, K. S. Plante, S. C. Weaver, P. Y. Shi, Spike mutation D614G alters SARS-CoV-2 fitness. *Nature* **592**, 116–121 (2021). doi:10.1038/s41586-020-2895-3 Medline
31. H. Tegally, E. Wilkinson, M. Giovanetti, A. Iranzadeh, V. Fonseca, J. Giandhari, D. Doolabh, S. Pillay, E. J. San, N. Msomi, K. Mlisana, A. von Gottberg, S. Walaza, M. Allam, A. Ismail, T. Mohale, A. J. Glass, S. Engelbrecht, G. Van Zyl, W. Preiser, F. Petruccione, A. Sigal, D. Hardie, G. Marais, M. Hsiao, S. Korsman, M.-A. Davies, L. Tyers, I. Mudau, D. York, C. Maslo, D. Goedhals, S. Abrahams, O. Laguda-Akingba, A. Alisoltani-Dehkordi, A. Godzik, C. K. Wibmer, B. T. Sewell, J. Lourenço, L. C. J. Alcantara, S. L. Kosakovsky Pond, S. Weaver, D. Martin, R. J. Lessells, J. N. Bhiman, C. Williamson, T. de Oliveira,Detection of a SARS-CoV-2 variant of concern in South Africa. *Nature* **592**, 438–443 (2021). doi:10.1038/s41586-021-03402-9
32. N. R. Faria, I. M. Claro, D. Candido, L. A. Moyses Franco, P. S. Andrade, T. M. Coletti, C. A. M. Silva, F. C. Sales, E. R. Manuli, R. S. Aguiar, N. Gaburo, C. da C. Camilo, N. A. Fraiji, M. A. Esashika Crispim, M. do Perpétuo S. S. Carvalho, A. Rambaut, N. Loman, O. G. Pybus, E. C. Sabino, on behalf of CADDE Genomic Network, "Genomic characterisation of an emergent SARS-CoV-2 lineage in Manaus: preliminary findings" (virological.org, 2021); https://virological.org/t/genomic-characterisation-of-an-emergent-sars-cov-2-lineage-in-manaus-preliminary-findings/586.
33. N. G. Davies, S. Abbott, R. C. Barnard, C. I. Jarvis, A. J. Kucharski, J. D. Munday, C. A. B. Pearson, T. W. Russell, D. C. Tully, A. D. Washburne, T. Wenseleers, A. Gimma, W. Waites, K. L. M. Wong, K. van Zandvoort, J. D. Silverman, K. Diaz-Ordaz, R. Keogh, R. M. Eggo, S. Funk, M. Jit, K. E. Atkins, W. J. Edmunds, Estimated transmissibility and impact of SARS-CoV-2 lineage B.1.1.7 in England. *Science* **372**, eabg3055 (2021). doi:10.1126/science.abg3055 Medline
34. D. A. Collier, A. De Marco, I. A. T. M. Ferreira, B. Meng, R. P. Datir, A. C. Walls, S. A. Kemp, J. Bassi, D. Pinto, C. Silacci-Fregni, S. Bianchi, M. A. Tortorici, J. Bowen, K. Culap, S. Jaconi, E. Cameroni, G. Snell, M. S. Pizzuto, A. F. Pellanda, C. Garzoni, A. Riva, A. Elmer, N. Kingston, B. Graves, L. E. McCoy, K. G. C. Smith, J. R. Bradley, N. Temperton, L. Ceron-Gutierrez, G. Barcenas-Morales, W. Harvey, H. W. Virgin, A. Lanzavecchia, L. Piccoli, R. Doffinger, M. Wills, D. Veesler, D. Corti, R. K. Gupta, Sensitivity of SARS-CoV-2 B.1.1.7 to mRNA vaccine-elicited antibodies. *Nature* **593**, 136–141 (2021). doi:10.1038/s41586-021-03412-7 Medline
35. P. Wang, M. S. Nair, L. Liu, S. Iketani, Y. Luo, Y. Guo, M. Wang, J. Yu, B. Zhang, P. D. Kwong, B. S. Graham, J. R. Mascola, J. Y. Chang, M. T. Yin, M. Sobieszczyk, C. A. Kyratsous, L. Shapiro, Z. Sheng, Y. Huang, D. D. Ho, Antibody resistance of SARS-CoV-2 variants B.1.351 and B.1.1.7. *Nature* **593**, 130–135 (2021). doi:10.1038/s41586-021-03398-2 Medline
36. S. A. Madhi, V. Baillie, C. L. Cutland, M. Voysey, A. L. Koen, L. Fairlie, S. D. Padayachee, K. Dheda, S. L. Barnabas, Q. E. Bhorat, C. Briner, G. Kwatra, K. Ahmed, P. Aley, S. Bhikha, J. N. Bhiman, A. E. Bhorat, J. du Plessis, A. Esmail, M. Groenewald, E. Horne, S.-H. Hwa, A. Jose, T. Lambe, M. Laubscher, M. Malahleha, M. Masenya, M. Masilela, S. McKenzie, K. Molapo, A. Moultrie, S. Oelofse, F. Patel, S. Pillay, S. Rhead, H. Rodel, L. Rossouw, C. Taoushanis, H. Tegally, A. Thombrayil, S. van Eck, C. K. Wibmer, N. M. Durham, E. J. Kelly, T. L. Villafana, S. Gilbert, A. J. Pollard, T. de Oliveira, P. L. Moore, A. Sigal, A. Izu; NGS-SA Group; Wits-VIDA COVID Group, Efficacy of the ChAdOx1 nCoV-19 Covid-19 vaccine against the B.1.351 variant. *N. Engl. J. Med.* **384**, 1885–1898 (2021). doi:10.1056/NEJMoa2102214 Medline
37. S. Cele, I. Gazy, L. Jackson, S.-H. Hwa, H. Tegally, G. Lustig, J. Giandhari, S. Pillay, E. Wilkinson, Y. Naidoo, F. Karim, Y. Ganga, K. Khan, M. Bernstein, A. B. Balazs, B. I. Gosnell, W. Hanekom, M. S. Moosa, R. J. Lessells, T. de Oliveira, A. Sigal; Network for Genomic Surveillance in South Africa; COMMIT-KZN Team, Escape of SARS-CoV-2 501Y.V2 from neutralization by convalescent plasma. *Nature* **593**, 142–146 (2021). doi:10.1038/s41586-021-03471-w Medline
38. D. Planas, T. Bruel, L. Grzelak, F. Guivel-Benhassine, I. Staropoli, F. Porrot, C. Planchais, J. Buchrieser, M. M. Rajah, E. Bishop, M. Albert, F. Donati, M. Prot, S. Behillil, V. Enouf, M. Maquart, M. Smati-Lafarge, E. Varon, F. Schortgen, L. Yahyaoui, M. Gonzalez, J. De Sèze, H. Péré, D. Veyer, A. Sève, E. Simon-Lorière, S. Fafi-Kremer, K. Stefic, H. Mouquet, L. Hocqueloux, S. van der Werf, T. Prazuck, O. Schwartz, Sensitivity of infectious SARS-CoV-2 B.1.1.7 and B.1.351 variants to neutralizing antibodies. *Nat. Med.* **27**, 917–924 (2021). doi:10.1038/s41591-021-01318-5 Medline
39. C. K. Wibmer, F. Ayres, T. Hermanus, M. Madzivhandila, P. Kgagudi, B. Oosthuysen, B. E. Lambson, T. de Oliveira, M. Vermeulen, K. van der Berg, T. Rossouw, M. Boswell, V. Ueckermann, S. Meiring, A. von Gottberg, C. Cohen, L. Morris, J. N. Bhiman, P. L. Moore, SARS-CoV-2 501Y.V2 escapes neutralization by South African COVID-19 donor plasma. *Nat. Med.* **27**, 622–625 (2021). doi:10.1038/s41591-021-01285-x Medline
40. X. Xie, Y. Liu, J. Liu, X. Zhang, J. Zou, C. R. Fontes-Garfias, H. Xia, K. A. Swanson, M. Cutler, D. Cooper, V. D. Menachery, S. C. Weaver, P. R. Dormitzer, P.-Y. Shi, Neutralization of SARS-CoV-2 spike 69/70 deletion, E484K and N501Y variants by BNT162b2 vaccine-elicited sera. *Nat. Med.* **27**, 620–621 (2021). doi:10.1038/s41591-021-01270-4 Medline
41. A. Rambaut, E. C. Holmes, Á. O'Toole, V. Hill, J. T. McCrone, C. Ruis, L. du Plessis, O. G. Pybus, A dynamic nomenclature proposal for SARS-CoV-2 lineages to assist genomic epidemiology. *Nat. Microbiol.* **5**, 1403–1407 (2020). doi:10.1038/s41564-020-0770-5 Medline

42. X. Deng, M. A. Garcia-Knight, M. M. Khalid, V. Servellita, C. Wang, M. K. Morris, A. Sotomayor-González, D. R. Glasner, K. R. Reyes, A. S. Gliwa, N. P. Reddy, C. Sanchez San Martin, S. Federman, J. Cheng, J. Balcerek, J. Taylor, J. A. Streithorst, S. Miller, B. Sreekumar, P.-Y. Chen, U. Schulze-Gahmen, T. Y. Taha, J. M. Hayashi, C. R. Simoneau, G. R. Kumar, S. McMahon, P. V. Lidsky, Y. Xiao, P. Hemarajata, N. M. Green, A. Espinosa, C. Kath, M. Haw, J. Bell, J. K. Hacker, C. Hanson, D. A. Wadford, C. Anaya, D. Ferguson, P. A. Frankino, H. Shivram, L. F. Lareau, S. K. Wyman, M. Ott, R. Andino, C. Y. Chiu, Transmission, infectivity, and neutralization of a spike L452R SARS-CoV-2 variant. *Cell* **184**, 3426–3437.e8 (2021). doi:10.1016/j.cell.2021.04.025 Medline
43. J. K. Millet, G. R. Whittaker, Murine leukemia virus (MLV)-based coronavirus spike-pseudotyped particle production and infection. *Bio Protoc.* **6**, e2035 (2016). doi:10.21769/BioProtoc.2035 Medline
44. T. N. Starr, N. Czudnochowski, F. Zatta, Y.-J. Park, Z. Liu, A. Addetia, D. Pinto, M. Beltramello, P. Hernandez, A. J. Greaney, R. Marzi, W. G. Glass, I. Zhang, A. S. Dingens, J. E. Bowen, J. A. Wojcechowskyj, A. De Marco, L. E. Rosen, J. Zhou, M. Montiel-Ruiz, H. Kaiser, H. Tucker, M. P. Housley, J. di Iulio, G. Lombardo, M. Agostini, N. Sprugasci, K. Culap, S. Jaconi, M. Meury, E. Dellota, E. Cameroni, T. I. Croll, J. C. Nix, C. Havenar-Daughton, A. Telenti, F. A. Lempp, M. S. Pizzuto, J. D. Chodera, C. M. Hebner, S. P. J. Whelan, H. W. Virgin, D. Veesler, D. Corti, J. D. Bloom, G. Snell, Antibodies to the SARS-CoV-2 receptor-binding domain that maximize breadth and resistance to viral escape. bioRxiv 438709 [Preprint]. 8 April 2021. https://doi.org/10.1101/2021.04.06.438709.
45. M. A. Tortorici, N. Czudnochowski, T. N. Starr, R. Marzi, A. C. Walls, F. Zatta, J. E. Bowen, S. Jaconi, J. di Iulio, Z. Wang, A. De Marco, S. K. Zepeda, D. Pinto, Z. Liu, M. Beltramello, I. Bartha, M. P. Housley, F. A. Lempp, L. E. Rosen, E. Dellota, H. Kaiser, M. Montiel-Ruiz, J. Zhou, A. Addetia, B. Guarino, K. Culap, N. Sprugasci, C. Saliba, E. Vetti, I. Giacchetto-Sasselli, C. S. Fregni, R. Abdelnabi, S.-Y. C. Foo, C. Havenar-Daughton, M. A. Schmid, F. Benigni, E. Cameroni, J. Neyts, A. Telenti, G. Snell, H. W. Virgin, S. P. J. Whelan, J. D. Bloom, D. Corti, D. Veesler, M. S. Pizzuto, Structural basis for broad sarbecovirus neutralization by a human monoclonal antibody. bioRxiv 438818 [Preprint]. 8 April 2021. https://doi.org/10.1101/2021.04.07.438818.
46. C. L. Hsieh, J. A. Goldsmith, J. M. Schaub, A. M. DiVenere, H. C. Kuo, K. Javanmardi, K. C. Le, D. Wrapp, A. G. Lee, Y. Liu, C. W. Chou, P. O. Byrne, C. K. Hjorth, N. V. Johnson, J. Ludes-Meyers, A. W. Nguyen, J. Park, N. Wang, D. Amengor, J. J. Lavinder, G. C. Ippolito, J. A. Maynard, I. J. Finkelstein, J. S. McLellan, Structure-based design of prefusion-stabilized SARS-CoV-2 spikes. *Science* **369**, 1501–1505 (2020). doi:10.1126/science.abd0826 Medline
47. K. McMahan, J. Yu, N. B. Mercado, C. Loos, L. H. Tostanoski, A. Chandrashekar, J. Liu, L. Peter, C. Atyeo, A. Zhu, E. A. Bondzie, G. Dagotto, M. S. Gebre, C. Jacob-Dolan, Z. Li, F. Nampanya, S. Patel, L. Pessaint, A. Van Ry, K. Blade, J. Yalley-Ogunro, M. Cabus, R. Brown, A. Cook, E. Teow, H. Andersen, M. G. Lewis, D. A. Lauffenburger, G. Alter, D. H. Barouch, Correlates of protection against SARS-CoV-2 in rhesus macaques. *Nature* **590**, 630–634 (2021). doi:10.1038/s41586-020-03041-6 Medline
48. P. S. Arunachalam, A. C. Walls, N. Golden, C. Atyeo, S. Fischinger, C. Li, P. Aye, M. J. Navarro, L. Lai, V. V. Edara, K. Röltgen, K. Rogers, L. Shirreff, D. E. Ferrell, S. Wrenn, D. Pettie, J. C. Kraft, M. C. Miranda, E. Kepl, C. Sydeman, N. Brunette, M. Murphy, B. Fiala, L. Carter, A. G. White, M. Trisal, C.-L. Hsieh, K. Russell-Lodrigue, C. Monjure, J. Dufour, S. Spencer, L. Doyle-Meyers, R. P. Bohm, N. J. Maness, C. Roy, J. A. Plante, K. S. Plante, A. Zhu, M. J. Gorman, S. Shin, X. Shen, J. Fontenot, S. Gupta, D. T. O'Hagan, R. Van Der Most, R. Rappuoli, R. L. Coffman, D. Novack, J. S. McLellan, S. Subramaniam, D. Montefiori, S. D. Boyd, J. A. L. Flynn, G. Alter, F. Villinger, H. Kleanthous, J. Rappaport, M. S. Suthar, N. P. King, D. Veesler, B. Pulendran, Adjuvanting a subunit COVID-19 vaccine to induce protective immunity. *Nature* **594**, 253–258 (2021). doi:10.1038/s41586-021-03530-2 Medline
49. A. Baum, D. Ajithdoss, R. Copin, A. Zhou, K. Lanza, N. Negron, M. Ni, Y. Wei, K. Mohammadi, B. Musser, G. S. Atwal, A. Oyejide, Y. Goez-Gazi, J. Dutton, E. Clemmons, H. M. Staples, C. Bartley, B. Klaffke, K. Alfson, M. Gazi, O. Gonzalez, E. Dick Jr., R. Carrion Jr., L. Pessaint, M. Porto, A. Cook, R. Brown, V. Ali, J. Greenhouse, T. Taylor, H. Andersen, M. G. Lewis, N. Stahl, A. J. Murphy, G. D. Yancopoulos, C. A. Kyratsous, REGN-COV2 antibodies prevent and treat SARS-CoV-2 infection in rhesus macaques and hamsters. *Science* **370**, 1110–1115 (2020). doi:10.1126/science.abe2402 Medline
50. Z. Liu, L. A. VanBlargan, L.-M. Bloyet, P. W. Rothlauf, R. E. Chen, S. Stumpf, H. Zhao, J. M. Errico, E. S. Theel, M. J. Liebeskind, B. Alford, W. J. Buchser, A. H. Ellebedy, D. H. Fremont, M. S. Diamond, S. P. J. Whelan, Identification of SARS-CoV-2 spike mutations that attenuate monoclonal and serum antibody neutralization. *Cell Host Microbe* **29**, 477–488.e4 (2021). doi:10.1016/j.chom.2021.01.014 Medline
51. Q. Li, J. Wu, J. Nie, L. Zhang, H. Hao, S. Liu, C. Zhao, Q. Zhang, H. Liu, L. Nie, H. Qin, M. Wang, Q. Lu, X. Li, Q. Sun, J. Liu, L. Zhang, X. Li, W. Huang, Y. Wang, The impact of mutations in SARS-CoV-2 spike on viral infectivity and antigenicity. *Cell* **182**, 1284–1294.e9 (2020). doi:10.1016/j.cell.2020.07.012 Medline
52. T. N. Starr, A. J. Greaney, A. S. Dingens, J. D. Bloom, Complete map of SARS-CoV-2 RBD mutations that escape the monoclonal antibody LY-CoV555 and its cocktail with LY-CoV016. *Cell Rep. Med.* **2**, 100255 (2021). doi:10.1016/j.xcrm.2021.100255 Medline
53. T. N. Starr, A. J. Greaney, S. K. Hilton, D. Ellis, K. H. D. Crawford, A. S. Dingens, M. J. Navarro, J. E. Bowen, M. A. Tortorici, A. C. Walls, N. P. King, D. Veesler, J. D. Bloom, Deep mutational scanning of SARS-CoV-2 receptor binding domain reveals constraints on folding and ACE2 binding. *Cell* **182**, 1295–1310.e20 (2020). doi:10.1016/j.cell.2020.08.012 Medline
54. V.-V. Edara, L. Lai, M. K. Sahoo, K. Floyd, M. Sibai, D. Solis, M. W. Flowers, L. Hussaini, C. R. Ciric, S. Bechnack, K. Stephens, E. B. Mokhtari, P. Mudvari, A. Creanga, A. Pegu, A. Derrien-Colemyn, A. R. Henry, M. Gagne, B. S. Graham, J. Wrammert, D. C. Douek, E. Boritz, B. A. Pinsky, M. S. Suthar, Infection and vaccine-induced neutralizing antibody responses to the SARS-CoV-2 B.1.617.1 variant. bioRxiv 443299 [Preprint]. 10 May 2021. https://doi.org/10.1101/2021.05.09.443299.
55. V. Tchesnokova, H. Kulakesara, L. Larson, V. Bowers, E. Rechkina, D. Kisiela, Y. Sledneva, D. Choudhury, I. Maslova, K. Deng, K. Kutumbaka, H. Geng, C. Fowler, D. Greene, J. Ralston, M. Samadpour, E. Sokurenko, Acquisition of the L452R mutation in the ACE2-binding interface of Spike protein triggers recent massive expansion of SARS-Cov-2 variants. bioRxiv 432189 [Preprint]. 11 March 2021. https://doi.org/10.1101/2021.02.22.432189.
56. E. C. Thomson, L. E. Rosen, J. G. Shepherd, R. Spreafico, A. da Silva Filipe, J. A. Wojcechowskyj, C. Davis, L. Piccoli, D. J. Pascall, J. Dillen, S. Lytras, N. Czudnochowski, R. Shah, M. Meury, N. Jesudason, A. De Marco, K. Li, J. Bassi, A. O'Toole, D. Pinto, R. M. Colquhoun, K. Culap, B. Jackson, F. Zatta, A. Rambaut, S. Jaconi, V. B. Sreenu, J. Nix, I. Zhang, R. F. Jarrett, W. G. Glass, M. Beltramello, K. Nomikou, M. Pizzuto, L. Tong, E. Cameroni, T. I. Croll, N. Johnson, J. Di Iulio, A. Wickenhagen, A. Ceschi, A. M. Harbison, D. Mair, P. Ferrari, K. Smollett, F. Sallusto, S. Carmichael, C. Garzoni, J. Nichols, M. Galli, J. Hughes, A. Riva, A. Ho, M. Schiuma, M. G. Semple, P. J. M. Openshaw, E. Fadda, J. K. Baillie, J. D. Chodera, S. J. Rihn, S. J. Lycett, H. W. Virgin, A. Telenti, D. Corti, D. L. Robertson, G. Snell; ISARIC4C Investigators; COVID-19 Genomics UK (COG-UK) Consortium, Circulating SARS-CoV-2 spike N439K variants maintain fitness while evading antibody-mediated immunity. *Cell* **184**, 1171–1187.e20 (2021). doi:10.1016/j.cell.2021.01.037 Medline
57. V. A. Avanzato, M. J. Matson, S. N. Seifert, R. Pryce, B. N. Williamson, S. L. Anzick, K. Barbian, S. D. Judson, E. R. Fischer, C. Martens, T. A. Bowden, E. de Wit, F. X. Riedo, V. J. Munster, Case study: Prolonged infectious SARS-CoV-2 shedding from an asymptomatic immunocompromised individual with cancer. *Cell* **183**, 1901–1912.e9 (2020). doi:10.1016/j.cell.2020.10.049 Medline
58. B. Choi, M. C. Choudhary, J. Regan, J. A. Sparks, R. F. Padera, X. Qiu, I. H. Solomon, H. H. Kuo, J. Boucau, K. Bowman, U. D. Adhikari, M. L. Winkler, A. A. Mueller, T. Y. Hsu, M. Desjardins, L. R. Baden, B. T. Chan, B. D. Walker, M. Lichterfeld, M. Brigl, D. S. Kwon, S. Kanjilal, E. T. Richardson, A. H. Jonsson, G. Alter, A. K. Barczak, W. P. Hanage, X. G. Yu, G. D. Gaiha, M. S. Seaman, M. Cernadas, J. Z. Li, Persistence and evolution of SARS-CoV-2 in an immunocompromised host. *N. Engl. J. Med.* **383**, 2291–2293 (2020). doi:10.1056/NEJMc2031364 Medline
59. K. R. McCarthy, L. J. Rennick, S. Nambulli, L. R. Robinson-McCarthy, W. G. Bain, G. Haidar, W. P. Duprex, Recurrent deletions in the SARS-CoV-2 spike glycoprotein drive antibody escape. *Science* **371**, 1139–1142 (2021). doi:10.1126/science.abf6950 Medline
60. E. Andreano, G. Piccini, D. Licastro, L. Casalino, N. V. Johnson, I. Paciello, S. D. Monego, E. Pantano, N. Manganaro, A. Manenti, R. Manna, E. Casa, I. Hyseni, L.

Benincasa, E. Montomoli, R. E. Amaro, J. S. McLellan, R. Rappuoli, SARS-CoV-2 escape in vitro from a highly neutralizing COVID-19 convalescent plasma. bioRxiv 424451 [Preprint]. 28 December 2020. https://doi.org/10.1101/2020.12.28.424451.
61. Y. Weisblum, F. Schmidt, F. Zhang, J. DaSilva, D. Poston, J. C. C. Lorenzi, F. Muecksch, M. Rutkowska, H.-H. Hoffmann, E. Michailidis, C. Gaebler, M. Agudelo, A. Cho, Z. Wang, A. Gazumyan, M. Cipolla, L. Luchsinger, C. D. Hillyer, M. Caskey, D. F. Robbiani, C. M. Rice, M. C. Nussenzweig, T. Hatziioannou, P. D. Bieniasz, Escape from neutralizing antibodies by SARS-CoV-2 spike protein variants. *eLife* **9**, e61312 (2020). doi:10.7554/eLife.61312 Medline
62. M. K. Annavajhala, H. Mohri, J. E. Zucker, Z. Sheng, P. Wang, A. Gomez-Simmonds, D. D. Ho, A.-C. Uhlemann, A novel SARS-CoV-2 variant, B.1.526, identified in New York. medRxiv 2021.02.23.21252259 [Preprint]. 12 June 2021. https://doi.org/10.1101/2021.02.23.21252259.
63. A. P. West Jr., C. O. Barnes, Z. Yang, P. J. Bjorkman, SARS-CoV-2 lineage B.1.526 emerging in the New York region detected by software utility created to query the spike mutational landscape. bioRxiv 431043 [Preprint]. 22 April 2021. https://doi.org/10.1101/2021.02.14.431043.
64. G. S. C. Slater, E. Birney, Automated generation of heuristics for biological sequence comparison. *BMC Bioinformatics* **6**, 31 (2005). doi:10.1186/1471-2105-6-31 Medline
65. K. Katoh, D. M. Standley, MAFFT multiple sequence alignment software version 7: Improvements in performance and usability. *Mol. Biol. Evol.* **30**, 772–780 (2013). doi:10.1093/molbev/mst010 Medline
66. D. Corti, J. Voss, S. J. Gamblin, G. Codoni, A. Macagno, D. Jarrossay, S. G. Vachieri, D. Pinna, A. Minola, F. Vanzetta, C. Silacci, B. M. Fernandez-Rodriguez, G. Agatic, S. Bianchi, I. Giacchetto-Sasselli, L. Calder, F. Sallusto, P. Collins, L. F. Haire, N. Temperton, J. P. Langedijk, J. J. Skehel, A. Lanzavecchia, A neutralizing antibody selected from plasma cells that binds to group 1 and group 2 influenza A hemagglutinins. *Science* **333**, 850–856 (2011). doi:10.1126/science.1205669 Medline
67. J. Gregson, S. Y. Rhee, R. Datir, D. Pillay, C. F. Perno, A. Derache, R. S. Shafer, R. K. Gupta, Human immunodeficiency virus-1 viral load is elevated in individuals with reverse-transcriptase mutation M184V/I during virological failure of first-line antiretroviral therapy and is associated with compensatory mutation L74I. *J. Infect. Dis.* **222**, 1108–1116 (2020). doi:10.1093/infdis/jiz631 Medline
68. X. Ou, Y. Liu, X. Lei, P. Li, D. Mi, L. Ren, L. Guo, R. Guo, T. Chen, J. Hu, Z. Xiang, Z. Mu, X. Chen, J. Chen, K. Hu, Q. Jin, J. Wang, Z. Qian, Characterization of spike glycoprotein of SARS-CoV-2 on virus entry and its immune cross-reactivity with SARS-CoV. *Nat. Commun.* **11**, 1620 (2020). doi:10.1038/s41467-020-15562-9 Medline
69. A. Takada, C. Robison, H. Goto, A. Sanchez, K. G. Murti, M. A. Whitt, Y. Kawaoka, A system for functional analysis of Ebola virus glycoprotein. *Proc. Natl. Acad. Sci. U.S.A.* **94**, 14764–14769 (1997). doi:10.1073/pnas.94.26.14764 Medline
70. A. M. Riblett, V. A. Blomen, L. T. Jae, L. A. Altamura, R. W. Doms, T. R. Brummelkamp, J. A. Wojcechowskyj, A haploid genetic screen identifies heparan sulfate proteoglycans supporting Rift Valley fever virus infection. *J. Virol.* **90**, 1414–1423 (2015). doi:10.1128/JVI.02055-15 Medline
71. K. H. D. Crawford, R. Eguia, A. S. Dingens, A. N. Loes, K. D. Malone, C. R. Wolf, H. Y. Chu, M. A. Tortorici, D. Veesler, M. Murphy, D. Pettie, N. P. King, A. B. Balazs, J. D. Bloom, Protocol and reagents for pseudotyping lentiviral particles with SARS-CoV-2 Spike protein for neutralization assays. *Viruses* **12**, 513 (2020). doi:10.3390/v12050513 Medline
72. A. C. Walls, B. Fiala, A. Schäfer, S. Wrenn, M. N. Pham, M. Murphy, L. V. Tse, L. Shehata, M. A. O'Connor, C. Chen, M. J. Navarro, M. C. Miranda, D. Pettie, R. Ravichandran, J. C. Kraft, C. Ogohara, A. Palser, S. Chalk, E. C. Lee, K. Guerriero, E. Kepl, C. M. Chow, C. Sydeman, E. A. Hodge, B. Brown, J. T. Fuller, K. H. Dinnon 3rd, L. E. Gralinski, S. R. Leist, K. L. Gully, T. B. Lewis, M. Guttman, H. Y. Chu, K. K. Lee, D. H. Fuller, R. S. Baric, P. Kellam, L. Carter, M. Pepper, T. P. Sheahan, D. Veesler, N. P. King, Elicitation of potent neutralizing antibody responses by designed protein nanoparticle vaccines for SARS-CoV-2. *Cell* **183**, 1367–1382.e17 (2020). doi:10.1016/j.cell.2020.10.043 Medline
73. J. B. Case, P. W. Rothlauf, R. E. Chen, N. M. Kafai, J. M. Fox, B. K. Smith, S. Shrihari, B. T. McCune, I. B. Harvey, S. P. Keeler, L. M. Bloyet, H. Zhao, M. Ma, L. J. Adams, E. S. Winkler, M. J. Holtzman, D. H. Fremont, S. P. J. Whelan, M. S. Diamond, Replication-competent vesicular stomatitis virus vaccine vector protects against SARS-CoV-2-mediated pathogenesis in mice. *Cell Host Microbe* **28**, 465–474.e4 (2020). doi:10.1016/j.chom.2020.07.018 Medline
74. C. Suloway, J. Pulokas, D. Fellmann, A. Cheng, F. Guerra, J. Quispe, S. Stagg, C. S. Potter, B. Carragher, Automated molecular microscopy: The new Leginon system. *J. Struct. Biol.* **151**, 41–60 (2005). doi:10.1016/j.jsb.2005.03.010 Medline
75. D. Tegunov, P. Cramer, Real-time cryo-electron microscopy data preprocessing with Warp. *Nat. Methods* **16**, 1146–1152 (2019). doi:10.1038/s41592-019-0580-y Medline
76. A. Punjani, H. Zhang, D. J. Fleet, Non-uniform refinement: Adaptive regularization improves single-particle cryo-EM reconstruction. *Nat. Methods* **17**, 1214–1221 (2020). doi:10.1038/s41592-020-00990-8 Medline
77. J. Zivanov, T. Nakane, B. O. Forsberg, D. Kimanius, W. J. Hagen, E. Lindahl, S. H. Scheres, New tools for automated high-resolution cryo-EM structure determination in RELION-3. *eLife* **7**, e42166 (2018). doi:10.7554/eLife.42166 Medline
78. J. Zivanov, T. Nakane, S. H. W. Scheres, A Bayesian approach to beam-induced motion correction in cryo-EM single-particle analysis. *IUCrJ* **6**, 5–17 (2019). doi:10.1107/S205225251801463X Medline
79. S. Chen, G. McMullan, A. R. Faruqi, G. N. Murshudov, J. M. Short, S. H. Scheres, R. Henderson, High-resolution noise substitution to measure overfitting and validate resolution in 3D structure determination by single particle electron cryomicroscopy. *Ultramicroscopy* **135**, 24–35 (2013). doi:10.1016/j.ultramic.2013.06.004 Medline
80. E. F. Pettersen, T. D. Goddard, C. C. Huang, G. S. Couch, D. M. Greenblatt, E. C. Meng, T. E. Ferrin, UCSF Chimera—A visualization system for exploratory research and analysis. *J. Comput. Chem.* **25**, 1605–1612 (2004). doi:10.1002/jcc.20084 Medline
81. P. Emsley, B. Lohkamp, W. G. Scott, K. Cowtan, Features and development of Coot. *Acta Crystallogr. D Biol. Crystallogr.* **66**, 486–501 (2010). doi:10.1107/S0907444910007493 Medline
82. R. Y. Wang, Y. Song, B. A. Barad, Y. Cheng, J. S. Fraser, F. DiMaio, Automated structure refinement of macromolecular assemblies from cryo-EM maps using Rosetta. *eLife* **5**, e17219 (2016). doi:10.7554/eLife.17219 Medline
83. B. Frenz, S. Rämisch, A. J. Borst, A. C. Walls, J. Adolf-Bryfogle, W. R. Schief, D. Veesler, F. DiMaio, Automatically fixing errors in glycoprotein structures with Rosetta. *Structure* **27**, 134–139.e3 (2019). doi:10.1016/j.str.2018.09.006 Medline
84. F. DiMaio, Y. Song, X. Li, M. J. Brunner, C. Xu, V. Conticello, E. Egelman, T. Marlovits, Y. Cheng, D. Baker, Atomic-accuracy models from 4.5-Å cryo-electron microscopy data with density-guided iterative local refinement. *Nat. Methods* **12**, 361–365 (2015). doi:10.1038/nmeth.3286 Medline
85. D. Liebschner, P. V. Afonine, M. L. Baker, G. Bunkóczi, V. B. Chen, T. I. Croll, B. Hintze, L. W. Hung, S. Jain, A. J. McCoy, N. W. Moriarty, R. D. Oeffner, B. K. Poon, M. G. Prisant, R. J. Read, J. S. Richardson, D. C. Richardson, M. D. Sammito, O. V. Sobolev, D. H. Stockwell, T. C. Terwilliger, A. G. Urzhumtsev, L. L. Videau, C. J. Williams, P. D. Adams, Macromolecular structure determination using X-rays, neutrons and electrons: Recent developments in Phenix. *Acta Crystallogr. D Struct. Biol.* **75**, 861–877 (2019). doi:10.1107/S2059798319011471 Medline
86. T. I. Croll, ISOLDE: A physically realistic environment for model building into low-resolution electron-density maps. *Acta Crystallogr. D Struct. Biol.* **74**, 519–530 (2018). doi:10.1107/S2059798318002425 Medline
87. V. B. Chen, W. B. Arendall 3rd, J. J. Headd, D. A. Keedy, R. M. Immormino, G. J. Kapral, L. W. Murray, J. S. Richardson, D. C. Richardson, MolProbity: All-atom structure validation for macromolecular crystallography. *Acta Crystallogr. D Biol. Crystallogr.* **66**, 12–21 (2010). doi:10.1107/S0907444909042073 Medline
88. B. A. Barad, N. Echols, R. Y. Wang, Y. Cheng, F. DiMaio, P. D. Adams, J. S. Fraser, EMRinger: Side chain-directed model and map validation for 3D cryo-electron microscopy. *Nat. Methods* **12**, 943–946 (2015). doi:10.1038/nmeth.3541 Medline
89. J. Agirre, J. Iglesias-Fernández, C. Rovira, G. J. Davies, K. S. Wilson, K. D. Cowtan, Privateer: Software for the conformational validation of carbohydrate structures. *Nat. Struct. Mol. Biol.* **22**, 833–834 (2015). doi:10.1038/nsmb.3115 Medline
90. T. D. Goddard, C. C. Huang, E. C. Meng, E. F. Pettersen, G. S. Couch, J. H. Morris, T. E. Ferrin, UCSF ChimeraX: Meeting modern challenges in visualization and analysis. *Protein Sci.* **27**, 14–25 (2018). doi:10.1002/pro.3235 Medline

ACKNOWLEDGMENTS

We thank Hideki Tani (University of Toyama) for providing the reagents necessary for preparing VSV pseudotyped viruses. This study was supported by the National Institute of Allergy and Infectious Diseases (DP1AI158186 and HHSN272201700059C to D.V., and U01 AI151698-01 to WCVV), a Pew Biomedical Scholars Award (D.V.), Investigators in the Pathogenesis of Infectious Disease Awards from the Burroughs Wellcome Fund (D.V.), Fast Grants (D.V.), the Natural Sciences and Engineering Research Council of Canada (M.M.), the Pasteur Institute (M.A.T). **Author contributions:** Conceived study: L.P., D.C., D.V. Designed study and experiments: M.M., J.B., A.D.M, A.C., A.C.W., J.d.I., M.A.T. Performed mutagenesis for mutant expression plasmids: M.M., E.C. and K.C. Performed mutant expression: M.M., J.E.B., E.C. and S.J. Contributed to donor's recruitment and plasma samples collection: S.B.G., G.B., A.F.P, C.G., S.T., W.V. Produced pseudoviruses and carried out pseudovirus neutralization assays. A.C.W., M.A.T., M.J.N., J.B., A.D.M., D.P., C.S., C.S-F. Bioinformatic analysis: J.d.I and A.T. Analyzed the data and prepared the manuscript with input from all authors: M.M., J.B., A.D.M., A.C.W., L.E.R., G.S., L.P., D.C. and D.V; supervision: M.S.P., L.P., G.S., H.W.V., D.C., and D.V. **Competing interests:** A.D.M., J.B., A.C., J.d.I., C.S-F., C.S., M.A., D.P., K.C., S.B., S.J., E.C., M.S.P., L.E.R., G.S., A.T., H.W.V., L.P. and D.C. are employees of Vir Biotechnology Inc. and may hold shares in Vir Biotechnology Inc. D.C. is currently listed as an inventor on multiple patent applications, which disclose the subject matter described in this manuscript. H.W.V. is a founder of PierianDx and Casma Therapeutics. Neither company provided funding for this work or is performing related work. D.V. is a consultant for Vir Biotechnology Inc. The Veesler laboratory has received a sponsored research agreement from Vir Biotechnology Inc. The remaining authors declare that the research was conducted in the absence of any commercial or financial relationships that could be construed as a potential conflict of interest. **Data and materials availability:** The cryoEM map and coordinates have been deposited to the Electron Microscopy Databank and Protein Data Bank with accession numbers EMD-24236, EMD-24237, PDB 7N8H and PDB 7N8I (see table S4 for details). Materials generated in this study are available from the corresponding authors upon request, but may require a completed Materials Transfer Agreement signed with Vir Biotechnology. This work is licensed under a Creative Commons Attribution 4.0 International (CC BY 4.0) license, which permits unrestricted use, distribution, and reproduction in any medium, provided the original work is properly cited. To view a copy of this license, visit https://creativecommons.org/licenses/by/4.0/. This license does not apply to figures/photos/artwork or other content included in the article that is credited to a third party; obtain authorization from the rights holder before using such material.

SUPPLEMENTARY MATERIALS

science.sciencemag.org/cgi/content/full/science.abi7994/DC1
Materials and Methods
Figs. S1 to S9
Tables S1 to S6
References (*64*–*90*)
MDAR Reproducibility Checklist

31 March 2021; accepted 25 June 2021
Published online 1 July 2021
10.1126/science.abi7994

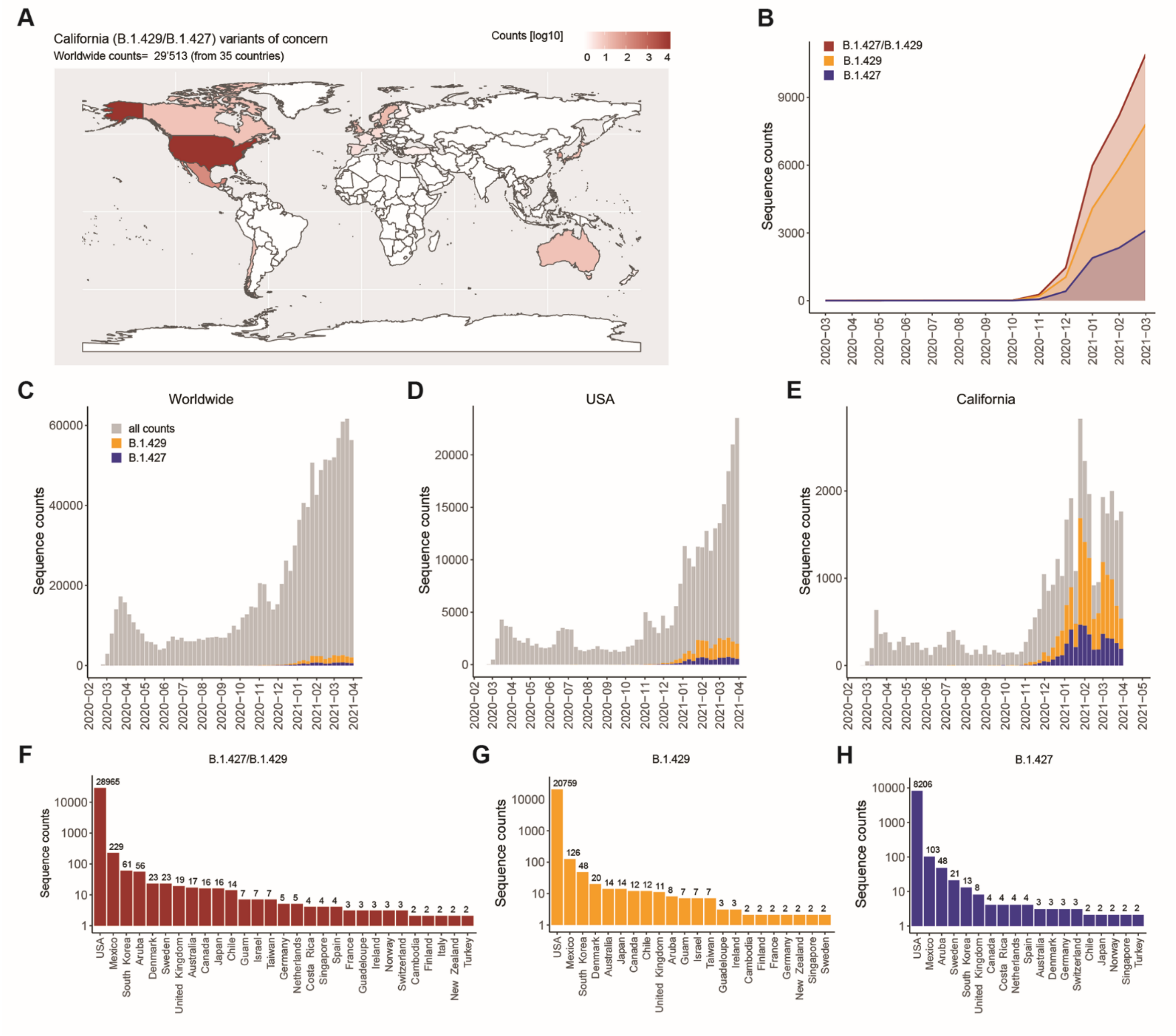

Fig. 1. Geographic distribution and evolution of incidence over time of the SARS-CoV-2 B.1.427/B.1.429 VOC. (**A**) World map showing the geographic distribution and sequence counts of B.1.427/B.1.429 VOC as of April 30, 2021. (**B**) Cumulative and individual B.1.427/B.1.429 VOC sequence counts by month. (**C** to **E**) Total number of SARS-CoV-2 (grey) and B.1.427/B.1.429 VOC (blue/orange) sequences deposited on a monthly basis worldwide (C), in the US (D) and in California (E). (**F** to **H**) Total number of B.1.427/B.1.429 (F), B.1.427 (G) and B.1.429 (H) sequences deposited by country as of April 30, 2021. Only countries with n≥2 deposited sequences are shown.

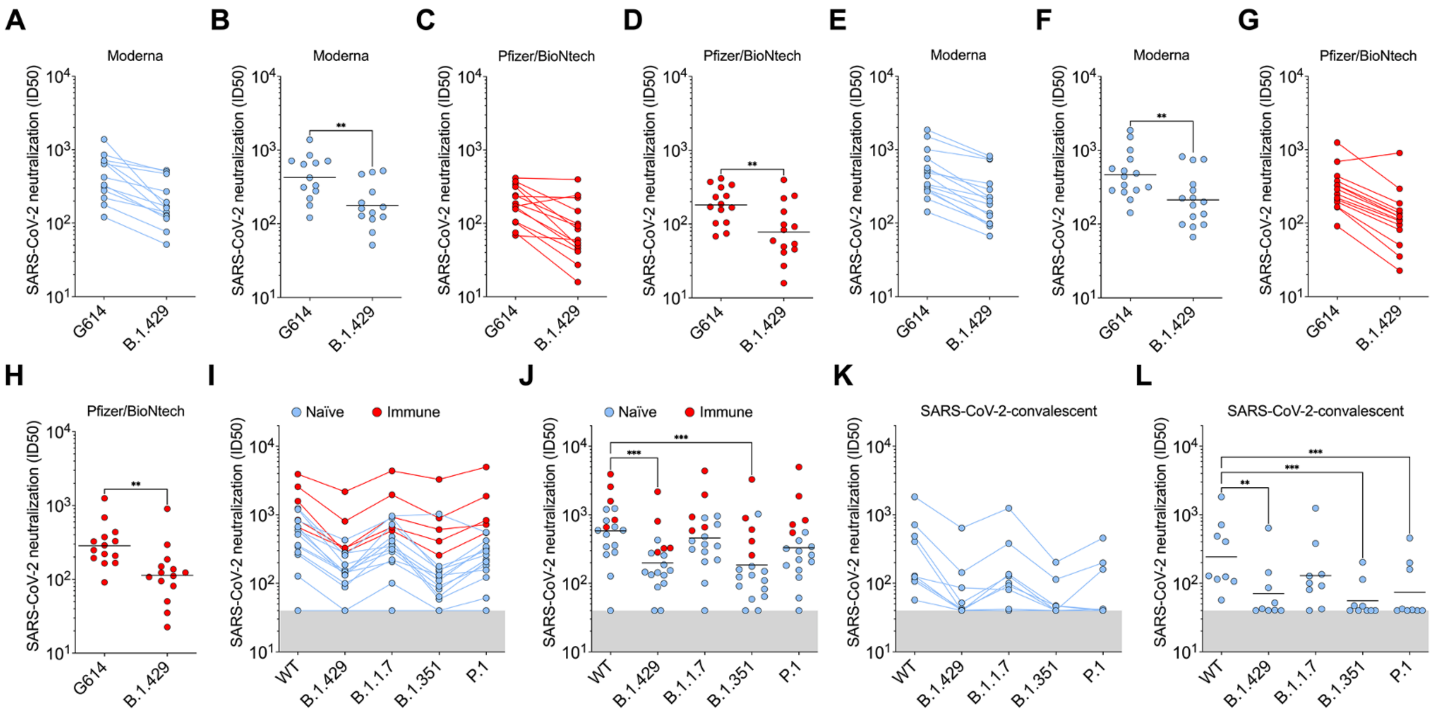

Fig. 2. B.1.427/B.1.429 S pseudotyped virus neutralization by vaccine-elicited and COVID-19 convalescent plasma. (**A**, **B**, E, and **F**) Neutralizing Ab titers (ID50) shown as pairwise connected [(A) and (E)] or the geometric mean titer, GMT [(B) and (F)] against MLV [(A) and (B)] or VSV [(E) and (F)] pseudotyped viruses harboring G614 SARS-CoV-2 S or B.1.427/B.1.429 (B.1.429) S determined using plasma from individuals who received two doses of Moderna mRNA-1273 vaccine (blue). (**C**, **D**, **G**, and **H**) Neutralizing Ab titers (ID50) shown as pairwise connected [(C) and (G)] or the geometric mean titer, GMT [(D) and (H)] against MLV [(C) and (D)] or VSV [(G) and (H)] pseudotyped viruses harboring G614 SARS-CoV-2 S or B.1.427/B.1.429 (B.1.429) S determined using plasma from individuals who received two doses of Pfizer/BioNtech BNT162b2 mRNA vaccine (red). (**I** and **J**) Neutralizing Ab ID50 (I) and GMT (J) titers against VSV pseudotyped viruses harboring D614 SARS-CoV-2 S, B.1.427/B.1.429 S, B.1.1.7 S, B.1.351 S, or P.1 S determined using plasma from naïve (blue) and previously infected (red) individuals who received two doses of Pfizer/BioNtech BNT162b2 mRNA vaccine. Naïve: vaccinated individuals who had not been previously infected with SARS-CoV-2. Immune: vaccinated individuals who had been previously infected with SARS-CoV-2. (**K** and **L**) Neutralizing Ab ID50 (K) and GMT (L) titers against VSV pseudotyped viruses harboring D614 SARS-CoV-2 S, B.1.427/B.1.429 S, B.1.1.7 S, B.1.351 S or P.1 S determined using plasma from convalescent individuals who were infected with wildtype SARS-CoV-2. Neutralization data shown in (A) to (H) and (I) to (L) were performed using 293T-ACE2 and VeroE6-TMPRSS2, respectively. Data are average of *n = 2* replicates.

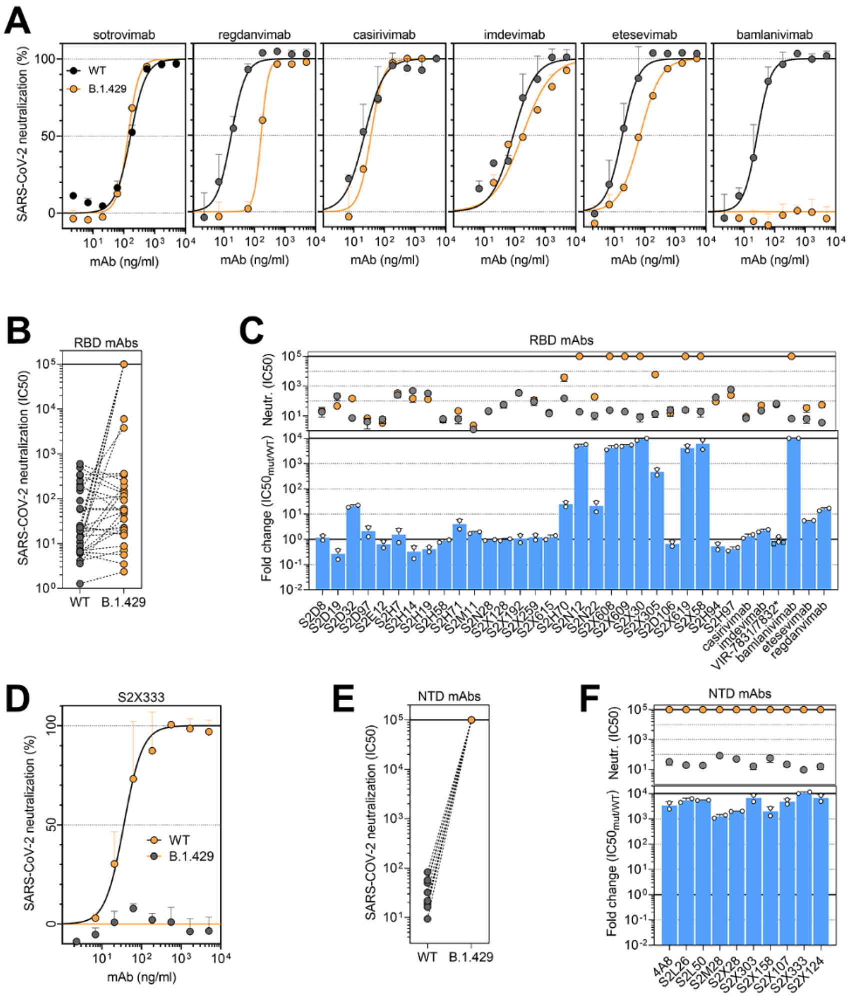

Fig. 3. Neutralization by a panel of RBD- and NTD-specific mAbs against SARS-CoV-2 D614 S and B.1.427/B.1.429 2 S pseudoviruses. (**A** and **D**) Neutralization of SARS-CoV-2 pseudotyped VSV carrying D614 (grey) or B.1.427/B.1.429 (orange) S protein by clinical-stage RBD mAbs (A) and an NTD-targeting mAb (S2X333) (D). Data are representative of $n = 2$ replicates. (**B** and **E**) Neutralization of SARS-CoV-2 S VSV pseudotypes carrying D614 or B.1.427/B.1.429 S by 34 mAbs targeting the RBD and 10 mAbs targeting the NTD. Data are the mean of 50% inhibitory concentration (IC_{50}) values (ng/ml) of $n = 2$ independent experiments. Non-neutralizing IC_{50} titers were set at 10^5 ng/ml. (**C** and **F**) Neutralization by RBD-specific (C) and NTD-specific (G) mAbs showed as mean IC_{50} values (top) and mean fold change (bottom) for B.1.427/B.1.429 S (orange) relative to D614G S (grey) VSV pseudoviruses. VIR-7831 is a derivative of S309 mAb (sotrovimab). *, VIR-7832 (variant of VIR-7831 carrying the LS-GAALIE Fc mutations) shown as squares. Non-neutralizing IC_{50} titers and fold change were set to 10^5 ng/ml and 10^4, respectively.

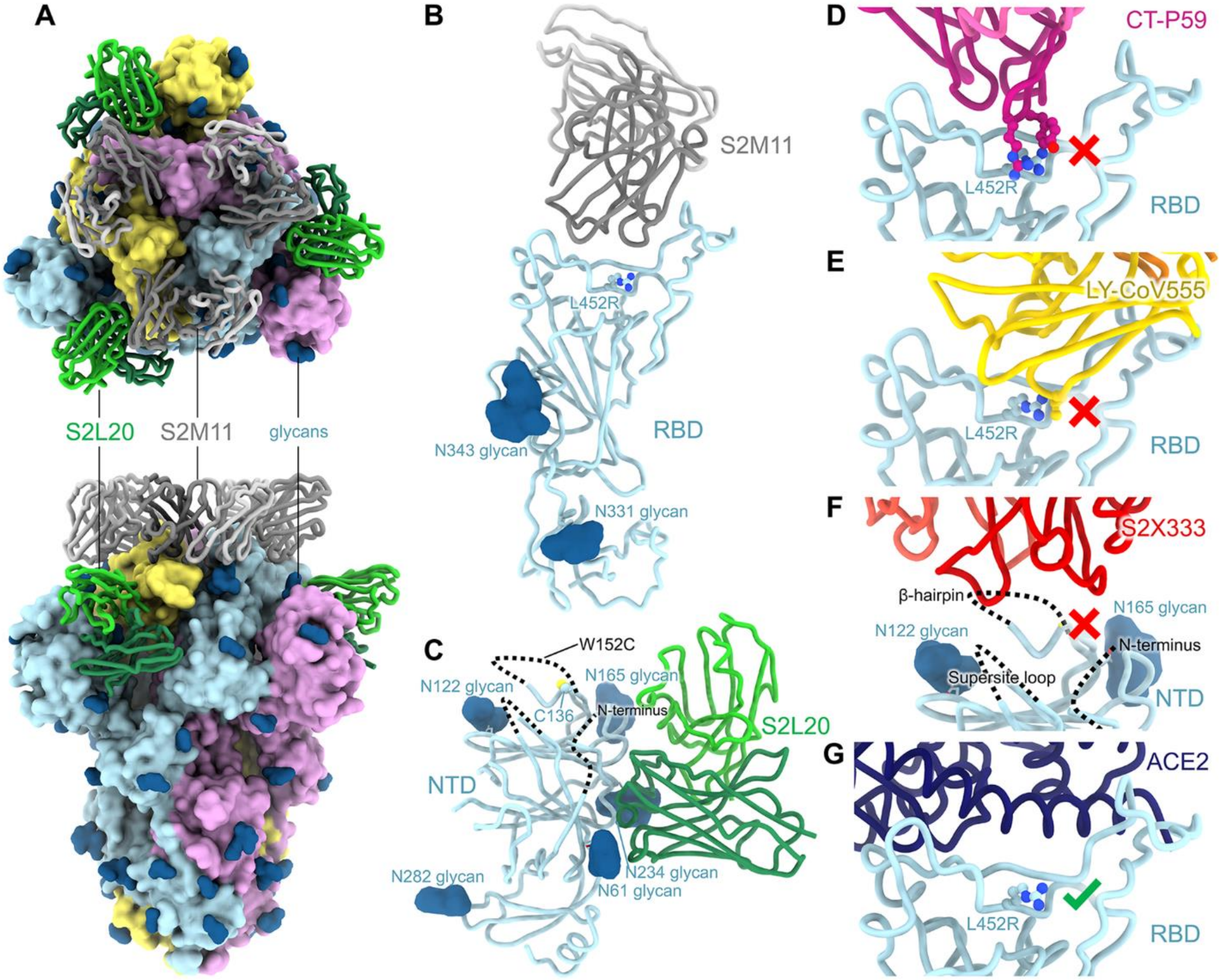

Fig. 4. CryoEM structure of the SARS-CoV-2 B.1.427/B.1.429 S ectodomain trimer. (**A**) Structure of the S trimer (surface rendering) bound to the S2M11 and S2L20 Fabs (ribbons) in two orthogonal orientations. SARS-CoV-2 S protomers are colored pink, cyan, and gold, whereas the S2L20 Fab heavy and light chains are colored dark and light green, respectively, and the S2M11 Fab heavy and light chains are colored dark and light gray, respectively. Only the Fab variable domains are resolved in the map. N-linked glycans are rendered as dark blue spheres. (**B**) Zoomed in view of the S2M11-bound RBD with R452 shown in ball and stick representation. (**C**) Zoomed in view of the S2L20-bound NTD with disordered N terminus, supersite β-hairpin and loop regions shown as dashed lines. (**D**) Superimposition of the CT-P59–bound SARS-CoV-2 RBD structure (PDB 7CM4) on the SARS-CoV-2 B.1.427/B.1.429 S cryoEM structure show that R452 would sterically clash with the mAb. (**E**) Superimposition of the LY-CoV555–bound SARS-CoV-2 RBD structure (PDB 7KMG) on the SARS-CoV-2 B.1.427/B.1.429 S cryoEM structure show that L452R would sterically clash with the mAb. (**F**) Superimposition of the S2X333-bound SARS-CoV-2 S structure (PDB 7LXW) on the SARS-CoV-2 B.1.427/B.1.429 S cryoEM structure reveals that most of the NTD antigenic supersite epitope residues are disordered. (**G**) Superimposition of the ACE2-bound SARS-CoV-2 RBD structure (PDB 7DMU) on the SARS-CoV-2 B.1.427/B.1.429 S cryoEM structure show that L452R points away from the interface with ACE2.

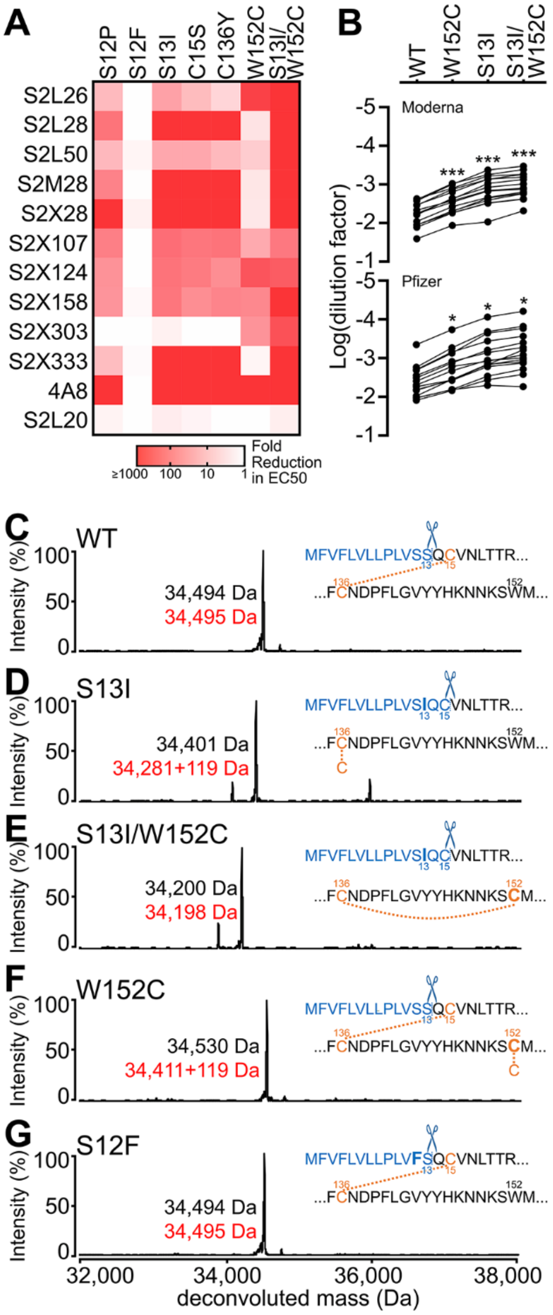

Fig. 5. The B.1.427/B.1.429 S S13I and W152C mutations lead to immune evasion. (**A**) Binding of a panel of 11 neutralizing (antigenic site i) and 1 non-neutralizing (antigenic site iv) NTD-specific mAbs to recombinant SARS-CoV-2 NTD variants analyzed by ELISA displayed as a heat map. (**B**) Binding of plasma Abs from vaccinated individuals to recombinant SARS-CoV-2 NTD variants analyzed by ELISA. The mean dilution factor for each mutant was compared by the one-way ANOVA test against wildtype yielding p values < 0.05 (*) and <0.001 (**). (**C** to **G**) Deconvoluted mass spectra of purified NTD constructs, including the wildtype NTD with the native signal peptide (B), the S13I NTD (C), the S13I and W152C NTD (D), the W152C NTD (E), and the S12F NTD (F). The empirical mass (black) and theoretical mass (red) are shown beside the corresponding peak. Additional 119 Da were observed for the S13I and W152C NTDs corresponding to cysteinylation of the free cysteine residue in these constructs (as L-cysteine was present in the expression media). The cleaved signal peptide (blue text) and subsequent residue sequence (black text) are also shown based on the MS results. Mutated residues are shown in bold. Cysteines are highlighted in light orange (unless in the cleaved signal peptide) while disulfide bonds are shown as dotted light orange lines between cysteines. Residues are numbered for reference.
